# Auranofin Synergizes with the PARP Inhibitor Olaparib to Induce ROS-Mediated Cell Death in Mutant p53 Cancers

**DOI:** 10.3390/antiox12030667

**Published:** 2023-03-08

**Authors:** Laurie Freire Boullosa, Jinthe Van Loenhout, Tal Flieswasser, Christophe Hermans, Céline Merlin, Ho Wa Lau, Elly Marcq, Marlies Verschuuren, Winnok H. De Vos, Filip Lardon, Evelien L. J. Smits, Christophe Deben

**Affiliations:** 1 Center for Oncological Research (CORE), Integrated Personalized & Precision Oncology Network (IPPON), University of Antwerp, 2610 Wilrijk, Belgium; 2 Laboratory of Cell Biology and Histology, Antwerp Center for Advanced Microscopy, Department of Veterinary Sciences, University of Antwerp, 2610 Wilrijk, Belgium; 3 µNEURO Research Centre of Excellence, University of Antwerp, 2610 Wilrijk, Belgium

**Keywords:** auranofin, olaparib, reactive oxygen species, non-small cell lung cancer, pancreatic ductal adenocarcinoma, cancer cell death

## Abstract

Auranofin (AF) is a potent, off-patent thioredoxin reductase (TrxR) inhibitor that efficiently targets cancer via reactive oxygen species (ROS)- and DNA damage-mediated cell death. The goal of this study is to enhance the efficacy of AF as a cancer treatment by combining it with the poly(ADP-ribose) polymerase-1 (PARP) inhibitor olaparib (referred to as ‘aurola’). Firstly, we investigated whether mutant p53 can sensitize non-small cell lung cancer (NSCLC) and pancreatic ductal adenocarcinoma (PDAC) cancer cells to AF and olaparib treatment in p53 knock-in and knock-out models with varying p53 protein expression levels. Secondly, we determined the therapeutic range for synergistic cytotoxicity between AF and olaparib and elucidated the underlying molecular cell death mechanisms. Lastly, we evaluated the effectiveness of the combination strategy in a murine 344SQ 3D spheroid and syngeneic in vivo lung cancer model. We demonstrated that high concentrations of AF and olaparib synergistically induced cytotoxicity in NSCLC and PDAC cell lines with low levels of mutant p53 protein that were initially more resistant to AF. The aurola combination also led to the highest accumulation of ROS, which resulted in ROS-dependent cytotoxicity of mutant p53 NSCLC cells through distinct types of cell death, including caspase-3/7-dependent apoptosis, inhibited by Z-VAD-FMK, and lipid peroxidation-dependent ferroptosis, inhibited by ferrostatin-1 and alpha-tocopherol. High concentrations of both compounds were also needed to obtain a synergistic cytotoxic effect in 3D spheroids of the murine lung adenocarcinoma cell line 344SQ, which was interestingly absent in 2D. This cell line was used in a syngeneic mouse model in which the oral administration of aurola significantly delayed the growth of mutant p53 344SQ tumors in 129S2/SvPasCrl mice, while either agent alone had no effect. In addition, RNA sequencing results revealed that AF- and aurola-treated 344SQ tumors were negatively enriched for immune-related gene sets, which is in accordance with AF’s anti-inflammatory function as an anti-rheumatic drug. Only 344SQ tumors treated with aurola showed the downregulation of genes related to the cell cycle, potentially explaining the growth inhibitory effect of aurola since no apoptosis-related gene sets were enriched. Overall, this novel combination strategy of oxidative stress induction (AF) with PARP inhibition (olaparib) could be a promising treatment for mutant p53 cancers, although high concentrations of both compounds need to be reached to obtain a substantial cytotoxic effect.

## 1. Introduction

Lung cancer and pancreatic ductal adenocarcinoma (PDAC) are the most common and lethal malignancies worldwide, with a 5-year overall survival rate of around 21% and 10%, respectively [1]. To date, treatment of these cancers remains challenging, partly due to their molecular signature in favor of the tumor, driven by the functional loss of multiple tumor suppressor genes, including *TP53* [2,3]. Therefore, there is an urgent unmet need for novel therapeutic approaches.

The p53 protein plays a crucial role as a tumor suppressor by regulating the cellular response to various stress signals and as the “guardian of the genome” by preventing genome mutations and the formation of malignant tumors [4]. However, p53’s normal function is often disturbed by inactivating or gain-of-function (GOF) mutations in the *TP53* gene, which are among the most common genetic lesions found in non-small cell lung cancer (NSCLC) [5] and PDAC [6], and result in the aberrant accumulation of mutant p53 protein and poor patient survival [7]. Mutant p53 GOF effects can influence the cellular redox balance, since it is able to suppress the activation and function of the major antioxidant transcription regulator NF-E2-related factor 2 (NRF2), leading to high intracellular reactive oxygen species (ROS) accumulation in cancer cells [7,8]. The latter makes mutant p53 cancer cells more sensitive towards oxidative stress induction. Contrary, normal cells can tolerate a certain level of exogenous ROS owing to their reserve antioxidant capacity and are capable of maintaining a normal redox balance [9]. Consequently, induction of oxidative stress and ROS could prove to be a highly selective anticancer approach in these cancer types [10,11].

A promising ROS-inducing compound is the thioredoxin reductase (TrxR) inhibitor auranofin (AF). AF was approved by the Food and Drug Administration (FDA) for the treatment of rheumatoid arthritis (RA) in the 1980′s which makes it an ideal candidate for drug repurposing in oncology [12,13]. Our previous research demonstrated the versatility and potency of AF as a clinically available, off-patent cancer drug that targets mutant p53 cancer cells through overall DNA damage and distinct ROS-mediated cell death pathways [14]. However, cancer cells can adapt to treatments targeting a single ROS-associated pathway by changing their metabolism or antioxidant defense system. Therefore, targeting cancer cells from different angles via a rational combination treatment offers an effective therapeutic opportunity to enhance AF’s anticancer effects, overcome tumor heterogeneity, and limit the ability of cancer cells to develop resistance [15].

An encouraging strategy is combining AF with a compound that inhibits DNA repair to counteract a possible route of cancer cell escape. AF-mediated generation of ROS and subsequent DNA damage activates the enzyme poly(ADP-ribose) polymerase-1 (PARP) in multiple cancer types [16,17,18,19,20,21]. PARP is a DNA damage sensor that plays a major role in DNA repair and in the maintenance of genomic stability [22]. To prevent repair, several compounds have been developed to inhibit the function of PARP, of which olaparib (AZD2281, Lynparza, AstraZeneca) is frequently used in the clinic [23]. Pharmacological inhibition of PARP is synthetically lethal, especially in cancer types with genetic or functional defects in *BRCA1/2* genes or other genes in the homologous recombination pathway [24]. In addition, it has already been shown by our group and others that PARP inhibition further sensitizes cancer cells to oxidative stress, highlighting the use of PARP inhibitors in combination with ROS-inducing compounds to induce synthetic lethality [22,25,26,27,28,29].

The presence of mutant p53 overexpression is an important sensitizer for AF treatment in NSCLC [14]. However, cells with lower mutant p53 protein levels were more resistant to AF treatment, and improvements in their response can still be made. Therefore, a selective combination strategy of oxidative stress induction and targeted PARP inhibition was explored in mutant p53 cancer cells. We hypothesized that AF, in combination with the PARP inhibitor olaparib, would induce a strong synergistic response both in vitro and in vivo with a high chance of successful implementation into the clinic. Since mutant p53 overexpression was already identified as an important sensitizer for AF in (isogenic) NSCLC and PDAC cells [14], we investigated the cytotoxic effect of AF and olaparib monotherapy in a NSCLC p53 knock-in and knock-out cell panel. In addition, we studied the cellular and cell death response of mutant p53 NSCLC and PDAC cell lines to the combination treatment of AF and olaparib (further referred to as ‘aurola’) in vitro. Our study revealed that the response to AF is synergistically enhanced by olaparib treatment in 2D mutant p53 NSCLC and PDAC cell cultures. Aurola-mediated cytotoxicity was dependent on high levels of ROS and killed mutant p53 NSCLC cells through distinct types of cell death, including apoptosis and ferroptosis. In a mutant p53 lung adenocarcinoma 129S2/SvPasCrl (129) mouse model, simultaneous delivery of AF and olaparib via oral gavage significantly reduced tumor growth compared to monotherapies. AF in the aurola combination treatment induced downregulation of immune-related pathways in vivo, while olaparib had a more beneficial effect on the immune system.

## 2. Materials and Methods

### 2.1. Human Cell Lines

A panel of eight human NSCLC and PDAC cell lines with differing p53 statuses was used in this study to cover a large subset of patients (Table S1). The human pancreatic cell lines included Mia-PaCa-2, Panc-1, Capan-2, and BxPC-3. The human NSCLC cell lines included NCI-H1975, NCI-H2228, NCI-H596, and A549. All these cell lines were purchased from the American Type Culture Collection, except for Mia-PaCa-2 (DSMZ-German Collection of Microorganisms and Cell Cultures). Three additional mutant p53 NSCLC cell line panels were used for most experiments performed in this study (Table S1). One panel was previously used by us [14] and consisted of the parental NSCLC NCI-H1299 cells (homozygous partial p53 deletion, Null) and its isogenic derivates stably transfected to express either mutant p53 R175H or R273H protein, as per established protocols [30] (kindly provided by prof. Dr. Haupt at the Peter MacCallum Cancer Centre, Australia). For the next panel, NCI-H2228 cells were stably transfected with three *TP53* shRNA viral vectors (shRNA1-3) to obtain mutant p53 knock-down and expanded as monoclonal isogenic cell lines as previously described [31]. A non-template control (NTC) was included. For the third cell panel, the wild-type (WT) p53 A549 cells were exposed to increasing concentrations of nutlin-3a over a period of 18 weeks. Afterward, monoclonal subclones were generated, of which the mutant p53 status was determined by next-generation sequencing (NGS) as described by Deben et al. [32]. For this study, the Y236N/R248W mutant p53 subclone A549.R2 was selected for further experiments.

Mia-PaCa-2, Panc-1, A549, and A549.R2 cells were cultured in DMEM (Life Technologies, Merelbeke, Belgium) supplemented with 10% fetal bovine serum (FBS, Life Technologies, Merelbeke, Belgium), 1% penicillin/streptomycin (Life Technologies), and 2 mM L-glutamine (Life Technologies, Merelbeke, Belgium). Capan-2, BxPC-3, NCI-H1975, NCI-H2228, NCI-H596 and the isogenic NCI-H1299 cell lines were cultured in RPMI (Life Technologies) supplemented as described above. Cells were grown as monolayers and maintained in an exponential growth phase in 5% CO_2_/95% air in a humidified incubator at 37 °C. All cell lines were confirmed as mycoplasma-free using the MycoAlert Mycoplasma Detection Kit (Lonza, Verviers, Belgium).

Separate batches of the NCI-H2228 and A549.R2 were stably transduced with the Incucyte NucLight Red Lentivirus reagent (Sartorius, Goettingen, Germany) with a puromycin resistance gene to allow cell tracking on the IncuCyte ZOOM system (Sartorius, Goettingen, Germany).

### 2.2. Murine Cell Line

The murine adenocarcinoma lung cancer cell line 344SQ derived from KrasLa1/+p53R172HΔG mice (subcutaneous metastasis) was a gift of Jonathan M. Kurie (The University of Texas, MD Anderson Cancer Center, Houston, TX, USA). This cell line is syngeneic to the male 129S2/SvPasCrl (129) mice. Cells were cultured in RPMI medium supplemented with 10% FBS and 10 mM L-glutamine. Cell lines were maintained at 37 °C and 5% CO_2_. Cells were tested on a routine base for mycoplasma contamination.

### 2.3. Sulforhodamine B Cytotoxicity Assay

A colometric sulforhodamine B (SRB) assay was used to measure treatment-induced cytotoxicity to AF and olaparib. The different cell lines were seeded in 96-well plates, incubated overnight, and exposed to 0–10 μM AF (in vitro experiments: MedChemExpress and Tocris; in vivo experiments: Sanbio) and 0–80 μM olaparib (AstraZeneca) for 72 h. Cell monolayers were then fixed with 10% trichloroacetic acid for 1 h at 4 °C and stained with 100 μL 0.1% SRB, as previously described [33]. The IC_50_ value (i.e., drug concentrations causing 50% growth inhibition) for every cell line was calculated using WinNolin Software (Pharsight, Mountain View, CA, USA).

### 2.4. Western Blotting

Cells were lysed in lysis buffer (10 mM TrisHCl, 400 mM NaCl, 1 mM EDTA, 0.1% NP40, and protease inhibitor). After centrifugation (10 min, 13,000 rpm, 4 °C), cleared lysates containing the isolated proteins were harvested and kept at −80 °C. Protein concentrations were determined using the Pierce BCA protein kit (Thermo Fisher, Brussels, Belgium) according to the manufacturer’s instructions. Western blotting for the detection of p53 and β-actin was performed as described previously [14].

### 2.5. Oxidative Stress Detection

Cells were seeded in 96-well plates, incubated overnight, and exposed to the correct concentrations of AF, olaparib, or aurola, alone or in combination with the ROS-scavenger N-acetyl-cysteine (NAC, 5 mM, Sigma-Aldrich, Schnelldorf, Germany). Immediately after treatment, 2.5 µM CellROX Green reagent (Invitrogen, Paisley, UK) was added to the cells. Afterward, the plate was transferred to the temperature- and CO_2_-controlled IncuCyte ZOOM (10× magnification). For analysis, the average green calibrated unit (GCU) was plotted for every cell line after 48 h for at least three repeats.

### 2.6. 2D Spark Cyto Cytotoxicity Assay

The optimal seeding density for each cell line was determined to ensure exponential growth for the entire duration of the assay. Cell suspensions were automatically seeded in 2D in black 384-well plates using the OT-2 pipetting robot (Opentrons) and allowed to settle overnight. Afterward, cells were treated with AF (0–6.5 μM), olaparib (0–30 μM), and their combination using the D300e digital drug dispenser (Tecan, Männedorf, Switzerland) with synergy wizard to treat cells with a 7-point drug titration matrix for each compound. Cell survival and cell death were assessed using an endpoint assay after 72 h. Therefore, cells were stained with 1000 nM Hoechst 33342 (blue) to determine the number of live cells and with 50 nM green-fluorescent marker Cytotox Green to measure the level of cell death in each well. The Tecan Spark Cyto live-cell imaging system was used to acquire images at 72 h to monitor drug response. The number of blue and green cells was counted using Tecan’s Image Analyzer Software. Potential synergism between AF and olaparib was determined using Synergy Finder (version 3.0) [34]. Synergy scores above 10 indicated synergism, scores between −10 and 10 implied an additive response, and those below −10 indicated an antagonistic effect.

### 2.7. IncuCyte ZOOM Analysis of Cell Death Pathways

NucLight Red transduced NCI-H2228 and A549.R2 cells were seeded in a 96-well plate. After overnight incubation, cells were preincubated with the desired cell death pathway inhibitors for 1 h (1 μM ferrostatin-1 (Fer1, Sigma-Aldrich), 100 μM alpha-tocopherol (αToco, Sigma-Aldrich), 10 μM Z-VAD-FMK (Bachem, Bubendorf, Switzerland), and 10 μM necrostatin-1 s (Nec-1s, Abcam, Cambridge, UK) or 4 h (100 μM Deferoxamine, DFO, Sigma-Aldrich). Then, NCI-H2228 cells were treated with 5 μM AF, 30 μM olaparib, and their combination, and A549.R2 cells were treated with 6.5 μM AF, 30 μM olaparib, and theirs combination in the presence of the IncuCyte Cytotox Green Reagent (50 nM, Sartorius) or Caspase-3/7 Green Apoptosis Reagent (2.5 μM, Sartorius). Afterward, the plate was transferred to the temperature- and CO_2_-controlled IncuCyte ZOOM for 72 h. Cell death was monitored by pictures (10× magnification) that were taken every 24 h to limit phototoxicity. For analysis, green object count (1/mm^2^), red object count (1/mm^2^), green-red overlapping object count (1/mm^2^) and Caspase-3/7 Green GCU × μm^2^/image were determined with the IncuCyte basic analyzer. The percentage of cell death and Caspase-3/7 positive cells were calculated with the formula: green object count/(red object count − overlapping object count) × 100.

### 2.8. Lipid Peroxidation

Cellular lipid ROS was measured using the Image-iT Lipid Peroxidation Kit (Invitrogen, Paisley, UK), according to the manufacturer’s instructions. Therefore, NCI-H2228 cells were treated with 5 μM AF, 30 μM olaparib, and their combination, and A549.R2 cells were treated with 6.5 μM AF, 30 μM olaparib, and their combination for 48 h or the positive control (100 μM cumene hydroperoxide) for 2 h. Afterward, 10 μM of the C11-BODIPY dye was added to the culture and incubated for 30 min at 37 °C. The acquisition was performed on a CytoFLEX (BD), and FlowJo v10.1 software (TreeStar) was used to calculate the ratios of C11-BODIPY red (590 nm) over green (520 nm) mean fluorescence intensity (MFI) signals.

### 2.9. Immunofluorescent Labeling

NCI-H2228 and A549.R2 cells were seeded in a black μClear 96-well plate, and after overnight incubation, they were treated with AF, olaparib, or their combination, aurola, for 48 h. Cultures were fixed in 2% PFA for 20 min at room temperature. Fixed cultures were permeabilized with 0.3% Triton X-100 in PBS (0.1 M, pH 7.4) for 8 min, followed by one-hour incubation with a primary antibody against the double-stranded break marker gamma-H2AX (γH2AX, 1:1000, Abcam) at RT in 50% FBS in PBS. After washing with PBS, secondary antibody GAM-488 (1:500, Invitrogen) was added for one hour at RT. Finally, DAPI was applied to the cultures for 20 min at a concentration of 5 μg/mL. Images were acquired with a Nikon Ti fluorescence microscope using a 20x dry lens (numerical aperture 0.75). Per well (technical replicate), 16 frames were acquired in three channels (395, 470 and 555 mm excitation). Image processing was performed in Fiji, a packaged version of ImageJ freeware [35]. The γH2AX spot occupancy (area fraction of the nucleus occupied by spots) was quantified using an image analysis pipeline that has been described before (https://github.com/DeVosLab/CellBlocks, accessed on 1 February 2023, Cellblocks.ijm) [14,36]. Nuclei were detected based on a DAPI counterstain using a trained convolutional neural network as implemented in the StarDist plugin [37]. Next, a Laplacian operator was used to selectively enhance γH2AX spots in the image prior to their detection with a user-defined threshold.

### 2.10. Generation and Culturing of 344SQ Spheroids

Spheroids were established from our murine adenocarcinoma lung cancer cell line 344SQ according to the following protocol. 344SQ cells were harvested with 0.05% trypsin-EDTA, and the desired volume of cell suspension was centrifuged at 1500 rpm at 4 °C. The cell pellet was resuspended in ice-cold 2/3 Cultrex Type 2 (R&D Systems) and 1/3 RPMI medium. Droplets of 20 μL, each containing 50,000 cells, were pipetted in a preheated 6-well culture plate. Next, plates were inverted and incubated for 30 min at 37 °C to solidify the Cultrex BME. Following incubation, prewarmed full RPMI medium was added on top of the droplets. Spheroids were allowed to grow for 3 days in 5% CO_2_/95% air in a humidified incubator at 37 °C before utilization in further experiments.

### 2.11. 3D Multi-Spheroid Cytotoxicity Assay

Three-day-old spheroids were harvested using Cultrex organoid harvesting solution and counted in a black 384-well plate using the Spark Cyto and in-house developed image analysis software Orbits [38]. Next, the spheroid seeding solution, consisting of the harvested 344SQ spheroids and 4% Cultrex Type 2, was automatically seeded under cold conditions in a precooled black 384-well plate at a concentration of 200 spheroids per well using the OT-2 pipetting robot. Afterward, spheroids were treated with AF monotherapy (0–5 μM), olaparib monotherapy (0–10 μM), or the combination of both (aurola) using the D300e digital drug dispenser. In addition, staurosporin (2 μM, Selleck Chemicals) was used as a positive cell death control, and the green-fluorescent marker Cytotox Green (50 nM) was added to measure the level of cell death in each well. Treated spheroid cultures were analyzed in real-time 72 h after treatment using the Orbits screening software [38], which quantified the total survival area (total brightfield area–total green area). The growth rate was normalized by defining the positive (staurosporin) and negative (untreated) control as 0% and 100% survival, respectively. Synergism between AF and olaparib was determined using Synergy Finder (version 3.0) [34]. The normalized drug response (NDR) was calculated as described by Gupta et al. [39]. NDR values above 1 indicated a growth-stimulatory effect, values between 0 and 1 indicated a cytostatic effect, and values below 0 implied a cytotoxic effect. Those viability values that barely changed and remained constant around 1 were classified as non-effective.

### 2.12. Mice

Male 129 mice, aged 6–9 weeks, were obtained from Charles River. All animal care and experimental procedures were approved by the Ethics Committee of the University of Antwerp (ECD code 2021-66). Upon arrival, mice were given a 7-day adaptation period before being used in experiments to reduce stress levels. All mice were housed in a temperature-controlled environment with 12-h light/dark cycles and received food and water ad libitum. Mice were checked on a daily basis to inspect their health and wellbeing.

### 2.13. Tumor Kinetics and Survival

Prior to injection, 344SQ cells were harvested using TrypLE (Gibco), washed three times with sterile PBS, and put through a 70-µm cell strainer to ensure a single-cell suspension without any contaminants. Next, mice were injected subcutaneously with 1 × 10^6^ 344SQ cells as previously described [40]. When tumors reached an average size of 40–50 mm^3^, mice were randomized based on tumor size and divided over four treatment groups (day 0): (1) vehicle control, (2) 15 mg/kg AF, (3) 200 mg/kg olaparib, and (4) 15 mg/kg AF + 200 mg/kg olaparib (aurola). Mice were given these treatments every day for two weeks via oral gavage using a 20G flexible feeding tube. AF was dissolved in a vehicle composed of 50% (*v*/*v*) DMSO, 40% (*v*/*v*) propylene glycol 300 (PEG300) and 10% absolute ethanol. Olaparib was formulated in 10% (*v*/*v*) DMSO and 50% (*v*/*v*) of 60% (*w*/*v*) HP-B-CD (Kleptose) in purified water. Tumor size was measured three times a week using a digital caliper. Tumor volume was calculated using the formula (length × width^2^)/2. Mice were sacrificed when a tumor size of 1500 mm^3^ was reached.

### 2.14. RNA Sequencing of Mice Tumors

For RNA sequencing (RNAseq), mice tumors were harvested on day 14 after treatment and transferred directly into RNAprotect Reagent (Qiagen, Antwerp, Belgium). Tumors were disrupted and homogenized using the TissueRuptor II (Qiagen). Afterward, RNA was extracted using the RNeasy midi kit (Qiagen) for tumor samples of 20–250 mg. For the removal of gDNA, RNAse-free DNAse treatment was performed. RNA concentration and purity were checked using the Qubit RNA BR Assay Kit on a Qubit 4 Fluorometer (Thermo Fisher) and NanoDrop ND-1000 (Thermo Fisher), respectively. Samples were frozen at −80 °C and delivered to Genomics Core Leuven for transcriptome sequencing using the Lexogen QuantSeq 3′ FWD library preparation kit for Illumina on a Hiseq400 SR50 line with a minimum of 2M reads per sample. Delivered read-count tables were analyzed using the online self-service analytics platform Omics Playground [41].

### 2.15. Statistics

Prism 9.0 software (GraphPad, Boston, MA, USA) was used for data comparison and graphical data representations. Statistical analyses were performed using SPSS Statistics 27 software (IBM, New York, NY, USA) and R, and significance for all statistics was reached if *p* ≤ 0.05. Spearman correlation coefficients were calculated to investigate the correlation between the different antioxidant-related proteins, olaparib IC_50_ values, and p53 protein levels in different NSCLC and PDAC cell lines. Unpaired non-parametric tests were performed to compare medians between two (Mann–Whitney U test) or multiple groups (Kruskal–Wallis test). Analysis of *γ*H2AX spot occupancy was performed using a non-parametric Kruskal–Wallis test with Dunn post hoc testing. To analyze differences in tumor kinetics over time, we used R [42] with the afex and emmeans [43] packages to perform mixed model ANOVAs. Differences in survival were analyzed using a log-rank test (Kaplan–Meier analysis).

## 3. Results

### 3.1. Effect of AF and Olaparib Monotherapy on Mutant p53 NSCLC and PDAC Cell Panel

**Figure 1:**
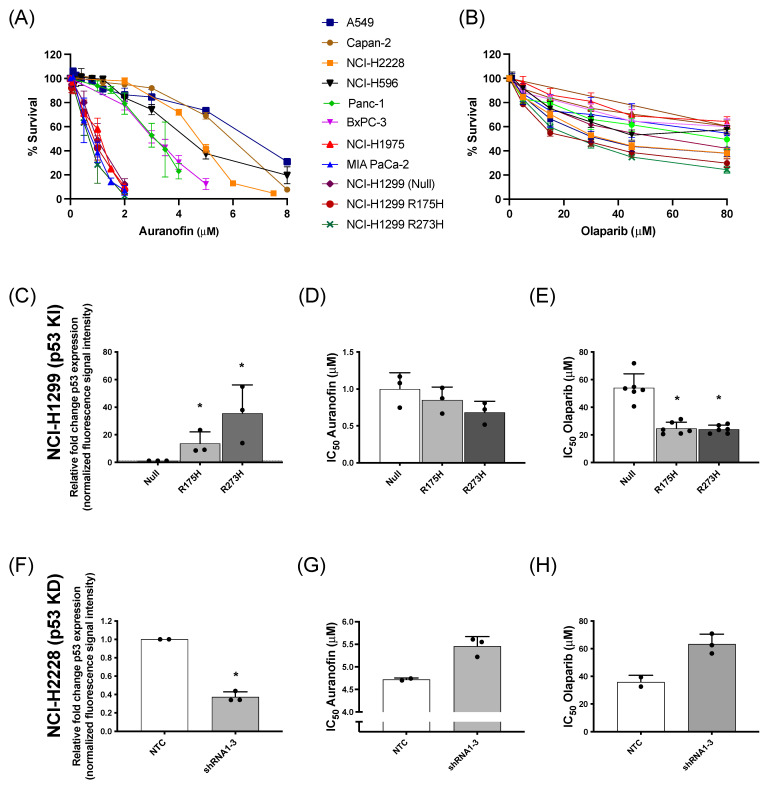
Cytotoxic response to AF and olaparib monotherapy in (isogenic) NSCLC and PDAC cell lines with distinct p53 backgrounds. (**A**,**B**) Dose-response survival curves after 72 h of AF (0–8 μM) (**A**) and olaparib treatment (0–80 μM) (**B**) in a panel of NSCLC and PDAC cell lines. (**C**) Fold change of p53 protein expression, relative to NCI-H1299 null control cells (determined by Western blotting), in the isogenic NCI-H1299 cell line panel. Representative Western blots are shown in Figure S2A. (**D**,**E**) IC_50_ values (μM) after 72 h of AF (**D**) or olaparib (**E**) treatment in the isogenic NCI–H1299 cell panel (determined by SRB assay). (**F**) Fold change of p53 protein expression, relative to NCI-H2228 NTC cells (determined by Western blotting), in the isogenic NCI-H2228 cells transfected with TP53 shRNA viral vector 1 to 3. Representative Western blots are shown in Figure S2B. (**G**,**H**) IC_50_ values (μM) after 72 h of AF (**G**) or olaparib (**H**) treatment in the isogenic NCI-H2228 cells transfected with either NTC or TP53 shRNA 1 to 3 (determined by SRB assay). Experiments were performed at least in duplicate with every dot representing a different repeat. Error bars represent the standard deviation. * *p* ≤ 0.05 significant differences. KI: knock-in p53; KD: knock-down p53; NTC: non-template control, parental cell line.

The cytotoxic effect of AF and olaparib monotherapy was investigated in a panel of NSCLC and PDAC cells with distinct p53 backgrounds and (mutant or WT) p53 protein levels (Table S1 and Figure S1A). Dose-response survival curves (Figure 1A) and their corresponding IC_50_ values (Figure S1B) showed variable sensitivity of the different cell lines to AF, which was inversely correlated with mutant p53 protein levels (Figure S1C). For olaparib treatment, the percentage of cell survival reached a plateau even at concentrations of 30 µM or higher (Figure 1B), with similar IC_50_ values among the different cell lines (Figure S1D).

Regarding the genetic background of these cell lines, our previous results identified the presence of high levels of mutant p53 protein as an important sensitizer for AF in (isogenic) NSCLC cells [14]. To further validate the role of mutant p53 in the response to AF and olaparib, both compounds were validated in NSCLC knock-in (NCI-H1299 Null, p53 R175H mutant vs. p53 R273H mutant) and NSCLC knock-down (NCI-H2228 NTC vs. *TP53* shRNA 1–3) cell panels of mutant p53 (Figure 1C–H). As previously shown by us [14], the p53 R273H mutant showed significantly higher levels of mutant p53 protein compared to R175H cells (Figure S2A) and was the most sensitive to AF (Figure 1C,D). Similarly, mutant p53 knock-in sensitized the isogenic NCI-H1299 cells to olaparib treatment (Figure 1E), suggesting a GOF effect of mutant p53. In addition, as expected, NTC NCI-H2228 cells displayed a significantly higher level of mutant p53 protein compared to the three NCI-H2228 subclones (shRNA 1–3) with *TP53* knock-down (Figure 1F and Figure S2B). Knock-down of *TP53* and consequently lower levels of mutant p53 protein increased the IC_50_ values of both AF and olaparib, making these cells less sensitive to both compounds (Figure 1G,H).

Overall, these data confirm that NSCLC and PDAC cells with high levels of mutant p53 protein are sensitive to AF [14], while cancer cells with lower mutant p53 protein levels are less sensitive to AF. In addition, higher levels of mutant p53 also increased the cytotoxic response to olaparib.

### 3.2. Aurola Treatment Leads to Synergistic ROS-Dependent Cytotoxicity in Mutant p53 NSCLC and PDAC Cell Lines with Low Mutant p53 Protein Levels

To investigate the potential interaction and synergy between AF and olaparib in more AF-resistant cancer cells with low mutant p53 and WT p53 protein levels (Figure S1A,B), 2D cell cultures of three NSCLC cell lines (NCI-H2228, A549 and A549.R2) and one PDAC cell line (BxPC3) were used to perform a broadly targeted titration of olaparib (1–30 μM), AF (0.5–6.5 μM), and their combination (aurola) using the Spark Cyto live-cell imaging system. These cell lines were selected based on their medium to low mutant p53 expression levels (Figure S1A) and, thereby, their poor response to AF monotherapy to determine if we could enhance the effect of AF using a combination treatment.

**Figure 2:**
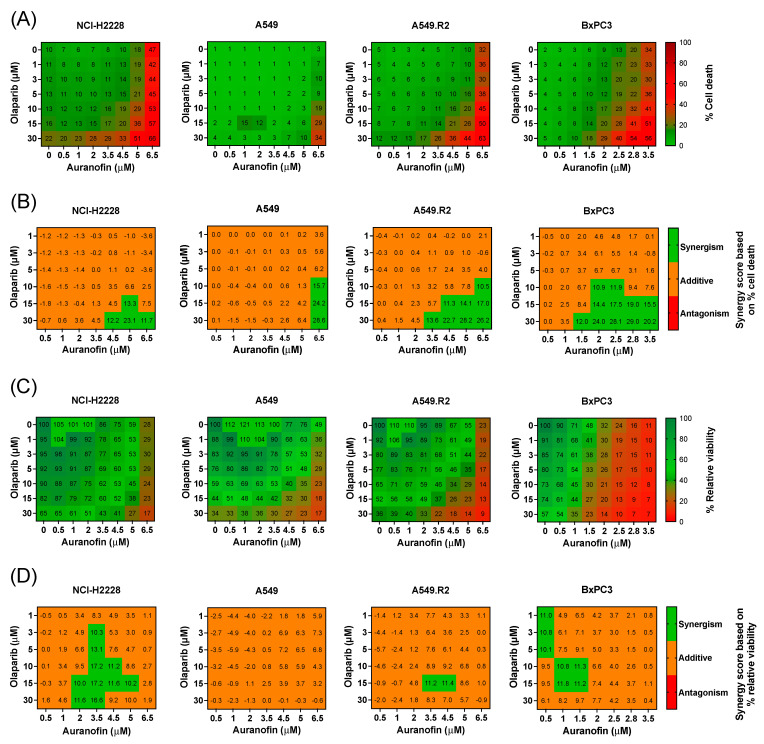
Cytotoxic multi-drug response to aurola treatment in NSCLC and PDAC cell lines in vitro. (**A**) Heatmap representing the cytotoxic effect of AF (0–6.5 µM) and olaparib (0–30 µM) monotherapy and their combinations after 72 h in the NSCLC cell lines NCI-H2888, A549, and A549.R2 and the PDAC cell line BxPC3. (**B**) Synergy scores for each concentration of AF and olaparib in the combination after 72 h of treatment in the NSCLC cell lines NCI-H2888, A549, and A549.R2 and the PDAC cell line BxPC3. Scores were based on drug combination response normalized to percentage inhibition (cell death). Synergy score > 10: synergistic interaction (green). Synergy score −10 to 10: additive interaction (orange). Synergy score < −10: antagonistic interaction (red) between two drugs. (**C**) Heatmap representing the relative viability of the NSCLC cell lines NCI-H2888, A549, and A549.R2 and the PDAC cell line BxPC3 after 72 h of treatment with AF (0–6.5 µM) and olaparib (0–30 µM) monotherapy and their combinations. (**D**) Synergy scores for each concentration of AF and olaparib in the combination after 72 h of treatment in the NSCLC cell lines NCI-H2888, A549, and A549.R2 and the PDAC cell line BxPC3. Scores were based on drug combination response normalized to percentage viability. Synergy score > 10: synergistic interaction (green). Synergy score −10 to 10: additive interaction (orange). Synergy score < −10: antagonistic interaction (red) between two drugs. Experiments were performed at least in triplicate. Representative images showing the effect of AF, olaparib, and aurola on the number of dead NSCLC and PDAC cells are shown in Figure S3.

Heatmaps representing percentage cell death (cytotoxicity) and percentage relative viability (survival) showed that mutant p53 cell lines (NCI-H2228, A549.R2, and BxPC3) were sensitive to mono- and combination treatments of AF and olaparib (Figure 2A,C and Figure S3). The higher the concentration of AF in the combination, the stronger their effect on the induction of cell death (Figure 2A). Since the PDAC cell line BxPC3 was the most sensitive, AF concentrations up to 3.5 µM were tested and compared to 6.5 µM for the other cell lines (Figure 2A–D). AF monotherapy induced significantly (*p* < 0.05) more cell death in the mutant p53 A549.R2 cells compared to the WT p53 A549 cells (Figure 2A and Figures S3 and S4). In addition, the aurola combination strategy was also much more effective in the A549.R2 cells, resulting in over 50% cell death in mutant p53 cells compared to 10% cell death in WT cells after combined treatment of 5 µM AF + 30 µM olaparib (*p* < 0.05) (Figure S4).

To quantify the synergistic cytotoxic effect, the online tool SynergyFinder (version 3.0) was used, and synergistic interactions were observed for the NSCLC cell lines at higher concentrations of AF (>3.5 μM) in combination with high concentrations of olaparib (>15 µM) (Figure 2B). For the more sensitive PDAC cell line BxPC3, synergistic interactions were observed between AF concentrations of 1.5 µM and higher with high concentrations of olaparib (>15 µM) (Figure 2B). When solely looking at relative cell viability, synergy was much less pronounced or absent (Figure 2D), indicating that aurola is a potent cytotoxic combination strategy rather than cytostatic.

**Figure 3:**
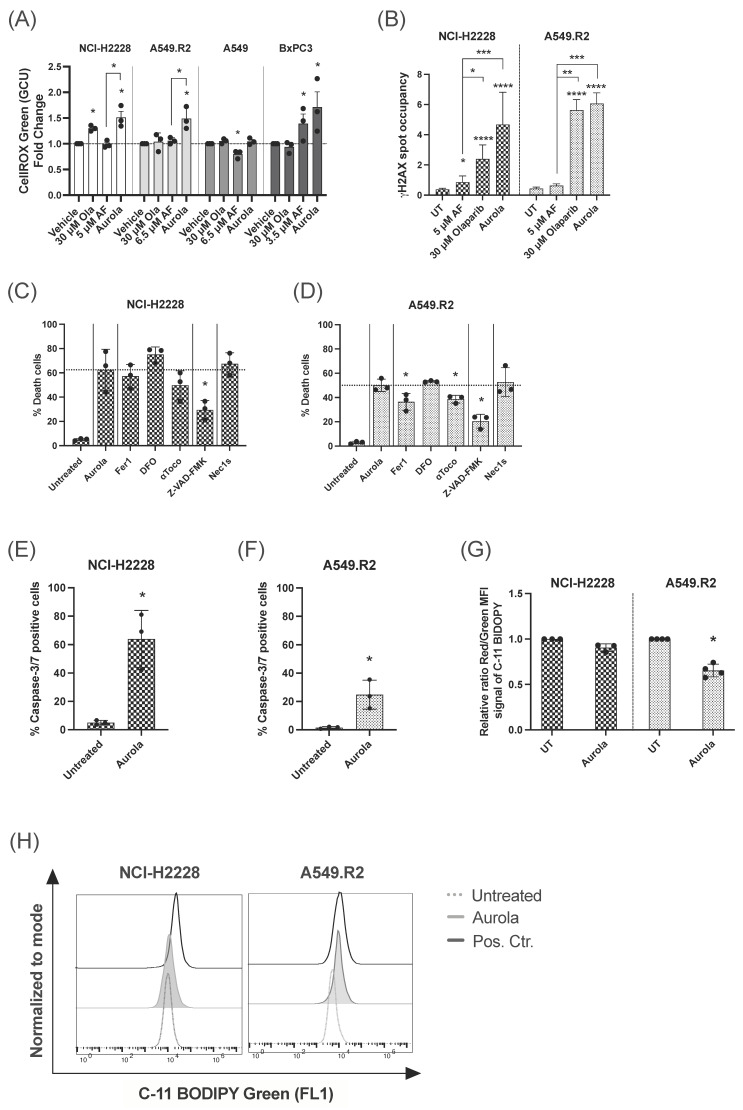
Induction of ROS-mediated apoptotic and ferroptotic cell death after aurola treatment of mutant p53 NSCLC cells. (**A**) Intracellular ROS levels shown as fold change of the CellROX Green Calibration Units (GCU) after 48 h of treatment with olaparib (30 μM), AF monotherapy (3.5–6.5 μM), and combination therapy, aurola. Experiments were performed in triplicate. (**B**) γH2AX spot occupancy calculated using an algorithm on Image J software in the NCI–H2228 and A549.R2 cell lines treated with PBS, 5 μM AF, 30 μM olaparib, and their combination for 48 h. Representative images are presented in Figure S7. Experiments were based on two biological repeats with six separate technical replicates per condition. (**C**,**D**) Percentage of death in NCI-H2228 cells (**C**) or death in A549.R2 cells (**D**) after treatment with aurola (5 μM AF + 30 μM olaparib or 6.5 μM AF + 30 μM olaparib, respectively) for 72 h in the absence or presence of apoptosis, necroptosis and ferroptosis inhibitors. (**E**,**F**) Percentage of caspase-3/7 green-positive cells after treatment of the NCI-H2228 (**E**) and A549.R2 cells (**F**) with aurola (5 μM AF + 30 μM olaparib or 6.5 μM AF + 30 μM olaparib, respectively) for 72 h. Error bars represent standard error of the mean. (**G**) Relative ratio of red over green mean fluorescent intensity (MFI) signal of the C11-BODIPY 581/591 reagent after treatment of NCI-H2228 and A549.R2 cells with aurola combination (5 μM AF + 30 μM olaparib or 6.5 μM AF + 30 μM olaparib, respectively) for 48 h or with cumene hydroperoxide (positive control) for two hours. (**H**) Overlay histograms of C11 BODIPY Green 581/591 signal after treatment of the NCI-H2228 and A549.R2 cells with aurola (5 μM AF + 30 μM olaparib or 6.5 μM AF + 30 μM olaparib, respectively) for 48 h or with cumene hydroperoxide for two hours. Experiments were performed in triplicate with every dot representing a different repeat. Error bars represent the standard deviation. * *p* ≤ 0.05, ** *p* ≤ 0.01, *** *p* ≤ 0.001, **** *p* ≤ 0.0001.

Quantitative analysis of ROS levels using the fluorescent sensor dye CellROX green revealed that the aurola combination treatment resulted in a strong upregulation of ROS levels in NCI-H2228, A549.R2, and BxPC3 cells, while AF monotherapy had a limited effect on the ROS levels in the NCI-H2228 and A549.R2 cells (Figure 3A). In A549 cells, AF monotherapy even significantly reduced the amount of cellular ROS (Figure 3A), indicating that in the presence of low levels of WT p53 protein, other antioxidant systems beside the Trx/TrxR system counteract AF-induced oxidative stress. To further investigate if ROS overproduction was involved in aurola-induced cell death, N-acetyl cysteine (NAC), a thiol-reducing antioxidant agent, was used to scavenge ROS. NAC pretreatment prevented the cytotoxic and synergistic effect of aurola in all cell lines, suggesting that excessive ROS formation is the prime cause of cell death induction by aurola (Figure S5A–D).

Based on the flattened and enlarged morphology of A549 cells after olaparib treatment shown in Figure S6, we studied the induction of senescence following olaparib treatment in the A549 and A549.R2 cells using the β-galactosidase assay. As expected, olaparib-treated A549 cells were visually positive for β-galactosidase, indicating that these cells were in a senescent state (Figure S6). However, in the mutant p53 A549.R2 cell line, olaparib failed to induce a senescent response (Figure S6). These findings further indicate the potential of mutant p53 as an important sensitizer for these treatments and that WT p53 is necessary for the induction of senescence following olaparib treatment.

These data show that the combination of AF and olaparib synergistically enhanced the induction of ROS-dependent cell death in NSCLC and PDAC 2D cell lines with lower levels of mutant p53 protein.

### 3.3. Aurola Treatment Results in Apoptotic and Ferroptotic Cell Death Characteristics in Mutant p53 NSCLC Cells

The high levels of ROS produced by aurola treatment in mutant p53 NCI-H2228 and A549.R2 cells (Figure 3A) can result in oxidative DNA damage [44,45]. Therefore, we hypothesized that by blocking PARP-mediated DNA repair via olaparib, DNA damage can accumulate in the cell in response to aurola-dependent ROS induction, resulting in cancer cell death. We observed a trend towards increased nuclear γH2AX spot occupancy after aurola treatment in the NCI-H2228 cells, indicating an increase in double-stranded breaks (Figure 3B and Figure S7). For the A549.R2 cells, this trend was less pronounced, whereby aurola did not result in more double-stranded DNA breaks compared to cells treated with olaparib monotherapy (Figure 3B and Figure S7).

Based on our previous results on the distinct AF-induced cell death mechanisms in mutant p53 NSCLC cells, we wanted to unravel the type of aurola-induced cell death in the mutant p53 NSCLC cell lines NCI-H2228 and A549.R2 using the same set of specific cell death pathway inhibitors [14]. The pan-caspase inhibitor Z-VAD-FMK significantly inhibited aurola-induced cell death in both cell lines compared to untreated cells (Figure 3C,D). Fluorescent imaging revealed a high percentage of caspase-3/7 positive NCI-H2228 cells after aurola treatment (Figure 3E), which indicated that aurola-mediated cell death of NCI-H2228 cells was induced through caspase-dependent apoptosis. To a smaller extent, aurola treatment induced a moderate increase in caspase-3/7-positive A549.R2 cells (Figure 3F). Concerning the latter, the aurola-induced cell death of A549.R2 cells was, next to Z-VAD-FMK, also partially inhibited by two of the three ferroptosis inhibitors Fer1 and αToco (Figure 3D). Ferroptosis is a type of programmed necrosis mainly triggered by lipid peroxidation arising from iron-dependent ROS accretion [46]. Ferroptosis was further supported by a significant increase in lipid peroxidation after treatment with aurola in the A549.R2 cell line but not the NCI-H2228 cell line (Figure 3G,H). The necroptosis inhibitor Nec-1s did not affect the percentage of cell death in both cell lines (Figure 3C,D).

Aurola treatment killed mutant p53 NSCLC cells through distinct types of cell death, including caspase-3/7-dependent apoptosis and lipid peroxidation-dependent ferroptosis. Apoptotic NCI-H2228 cells were more sensitive to aurola-induced double-stranded breaks and relative DNA damage compared to A549.R2 cells.

### 3.4. Clinically Relevant Concentrations of Olaparib Induce a Synergistic Effect in Combination with AF in 3D Cultures of the Murine Lung Adenocarcinoma Cell Line 344SQ

Before validating the aurola combination in vivo, we determined the sensitivity of a murine adenocarcinoma lung cancer cell line 344SQ to AF and olaparib in a 2D and 3D in vitro setting. Based on the recommendations of AstraZeneca, more clinically relevant concentrations of olaparib were tested via a broadly targeted titration of olaparib (0–10 µM) in combination with AF (0–5 µM) in both 2D 344SQ cells and 3D 344SQ spheroids using the Spark Cyto live-cell imaging system.

**Figure 4:**
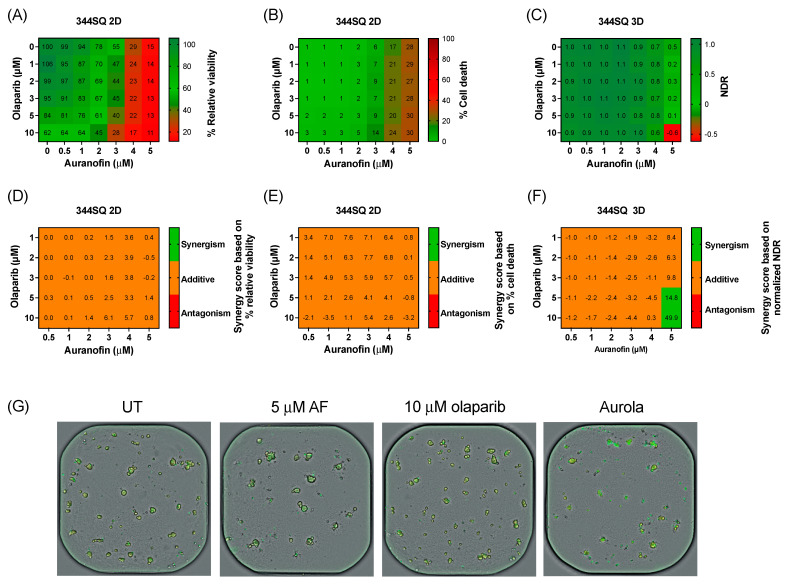
Cytotoxic effect of aurola at more clinically relevant concentrations in the murine 344SQ cells in 2D cultures and 3D spheroids. (**A**) Heatmap representing the relative viability of 2D 344SQ cells after 72 h of treatment with AF (0–5 µM) and olaparib (0–10 µM) monotherapy and their combinations. (**B**) Heatmap representing the cytotoxic effect of AF (0–5 µM) and olaparib (0–10 µM) monotherapy and their combinations after 72 h in 2D 344SQ cells. (**C**) Heatmap representing the normalized drug response (NDR) of AF (0–5 µM) and olaparib (0–10 µM) monotherapy and their combinations after 72 h in 3D 344SQ spheroids. (**D**) Synergy scores for each concentration of AF and olaparib in the targeted titration series after 72 h of treatment in 2D 344SQ cells. Scores were based on drug combination response normalized to percentage viability. (**E**) Synergy scores for each concentration of AF and olaparib in the targeted titration series after 72 h of treatment in 2D 344SQ cells. Scores were based on drug combination response normalized to percentage inhibition (cell death). (**F**) Synergy scores for each concentration of AF and olaparib in the targeted titration series after 72 h of treatment in 3D 344SQ spheroids. Scores were based on drug combination response normalized to NDR. Synergy score >10: synergistic interaction (green). Synergy score −10 to 10: additive interaction (orange). Synergy score < −10: antagonistic interaction (red) between two drugs. (**G**) Representative images of 3D 344SQ spheroids treated with corresponding concentrations of AF, olaparib, and their combination using the Spark Cyto imaging system. Yellow signal masks the 344SQ spheroids, while cytotox green signal visualizes dead spheroids. Experiments were performed at least in duplicate.

Therapy response in 2D 344SQ cell cultures was based on the percentage relative viability (survival) and cell death (cytotoxicity), while for the 3D spheroids, their response was determined via NDR. Olaparib monotherapy had little to no effect on the viability or cell death of 2D 344SQ cells and 3D spheroids (Figure 4A–C). A decrease in survival was observed after AF monotherapy and the combination in both 2D and 3D (Figure 4A,C). Using the online tool SynergyFinder on both survival and cell death data, only an additive effect was observed between AF and olaparib treatment among all concentrations of the titration series in the 2D 344SQ cells (Figure 4D,E). Interestingly, in 3D 344SQ spheroids, there was a strong synergistic interaction between the highest concentration of 5 µM AF and the highest concentrations of olaparib (5 and 10 µM) (Figure 4F), as shown in the representative graphs of Figure 4G.

Despite the use of more clinically realistic concentrations, high concentrations of both compounds were needed to obtain a substantial cytotoxic effect.

### 3.5. Aurola Delays Tumor Growth and Downregulates Immune-Related and Cell Cycle-Related Gene Sets in 344SQ Mice Tumors

We further investigated whether aurola treatment could lead to an augmented anti-tumor response in a syngeneic mutant p53 344SQ 129-mouse model. Since synergy was only observed at higher concentrations of AF and olaparib in vitro, we aimed to determine if sufficiently high concentrations of both AF and olaparib, based on the literature [40], could be achieved in vivo to obtain a synergistic anti-tumoral response.

**Figure 5:**
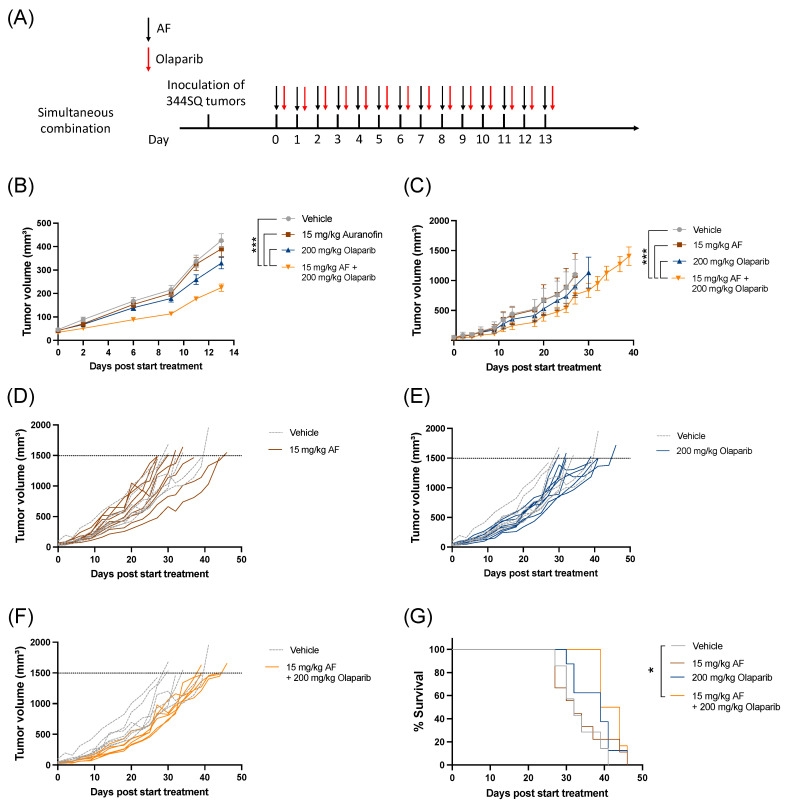
Tumor kinetics and survival after aurola combination therapy in syngeneic lung adenocarcinoma mouse model. Male 129S2/SvPasCrl (129-) mice were injected with 1 × 10^6^ 344SQ cells subcutaneously. When tumors reached a size of 40–50 mm^3^ (day 0), mice were randomized and treated with vehicle, AF (15 mg/kg), olaparib (200 mg/kg), or aurola (15 mg/kg AF + 200 mg/kg olaparib). (**A**) Treatment scheme showing timing of AF treatment (15 mg/kg via oral gavage administered for 14 consecutive days) with black arrows and olaparib treatment (200 mg/kg via oral gavage administered for 14 consecutive days) with red arrows. (**B**) Tumor growth kinetics until day 13 after different treatments as indicated (*n* = 12–14 mice per group). Error bars represent the standard error of the mean. (**C**) Tumor growth kinetics over time (*n* = 6–8 mice per group) after different treatments as indicated. Error bars represent the standard deviation. (**D**–**F**) Spaghetti plots of tumor volumes for individual mice in each treatment group (solid lines) compared to individual mice in vehicle group (dotted lines). (**G**) Survival of 129-mice (*n* = 6–8 mice per group) after different treatments as indicated. * *p* ≤ 0.05; *** *p* ≤ 0.001.

The combination of AF and olaparib was delivered orally for 14 consecutive days via a simultaneous treatment schedule (Figure 5A) without any side effects or weight loss (Figure S8). Aurola treatment induced a significant delay in tumor growth during 14 days of treatment (Figure 5B) and over time (Figure 5C–F) compared to both monotherapies and vehicle-treated mice. There was only a significant increase in survival after treatment with aurola compared to vehicle, but not with monotherapies (Figure 5G).

To unravel the in vivo effects caused by the single agents and their combination, RNAseq was performed on 344SQ tumors harvested on day 14 in the treatment schedule (Figure 5A) and analyzed using the Omics Playground platform. A UMAP-based clustering analysis showed that there was a substantial variability in expression between replicates within each treatment group. Combination-treated samples were clustered most distinctly from the vehicle-treated samples (Figure S9). Next, hierarchical clustering was performed for the top 150 differentially expressed genes split by treatment group into four gene clusters (Figure S10A), and cluster annotation was performed on the gene set level for the Hallmark Collection with Fisher’s test weighting (Figure S10B). The top-ranked annotation features include genes downregulated by KRAS activation in clusters S1 and S2 in the olaparib and combination group compared to the vehicle group. Genes related to angiogenesis were upregulated in the combination group (cluster S3). Finally, AF had a clear inverse effect on several immunologic gene sets, including interferon (IFN)-α and -γ response, as shown in cluster S4 (Figure S10A,B).

**Figure 6:**
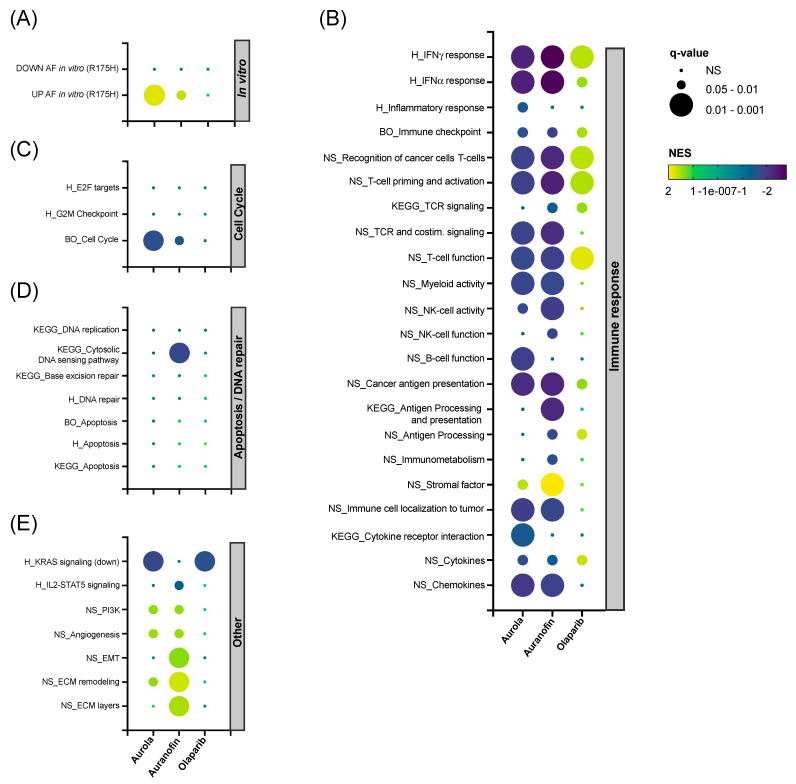
Effect of AF, olaparib and aurola treatment on immune-related, apoptosis, DNA repair and cell cycle gene sets in vivo. 344SQ tumors (*n* = 6–8 mice per group) were harvested on day 14 of the treatment schedule for subsequent RNA isolation and sequencing. (**A**–**E**) Bubble plots showing the effect of AF, olaparib, and aurola treatment on a selected panel of verified in-house, Nanostring (NS), BigOmics (BO), Hallmark (H) and KEGG gene sets in 344SQ mice tumors, including gene sets related to the in vitro response of mutant p53 R175H NCI-H1299 cells after AF treatment determined via RNA sequencing (**A**), immune response (**B**), cell cycle (**C**), apoptosis/DNA repair (**D**), and others (**E**). Bubble size corresponds to the degree of significance (q-value). Bubble color corresponds to positive NES (upregulated gene sets in yellow–green) and negative NES (downregulated gene sets in blue–purple). NES: normalized enrichment score; EMT: epithelial-to-mesenchymal transition; ECM: extracellular matrix.

Next, we performed gene set enrichment analysis (GSEA) on a selected panel of verified in-house, Nanostring, BigOmics and KEGG gene sets. Firstly, AF-treated mice samples were enriched for the top upregulated genes found in NCI-H1299 R175H cancer cells exposed to AF in vitro (Figure 6A) [14]. Secondly, both AF monotherapy and the combination therapy were negatively enriched for most immune-related gene sets involved in IFN-γ response and T cell function, while the inverse was observed for olaparib monotherapy (Figure 6B). Third, mice tumors treated with aurola showed the downregulation of genes involved in the cell cycle, which could potentially explain the inhibitory effect of the combination therapy on tumor growth (Figure 6C). In contrast with the in vitro data (Figure 3B–F), no apoptotic or DNA repair pathways were affected in the tumor after aurola treatment (Figure 6D). Finally, AF monotherapy samples were enriched for genes related to epithelial-to-mesenchymal transition (EMT), extracellular matrix (ECM) remodeling, and ECM layers, which was nullified in the combination strategy with olaparib (Figure 6E).

## 4. Discussion

The cornerstone of cancer treatment is to eradicate irresponsive and resistant cancer cells through the development of new combination strategies that can tackle different hallmarks of cancer cells. One of these hallmarks is increased oxidative stress, which makes cancer cells more vulnerable to the induction of ROS [47]. Our previous results revealed that the TrxR inhibitor AF triggered distinct ROS-mediated cell death mechanisms and overall DNA damage in mutant p53 NSCLC cells [14]. However, improvements are needed for cancer cells that are more resistant to AF monotherapy and to reach more clinically relevant concentrations via a well-designed combination strategy. As a result, we selected the PARP inhibitor olaparib as a promising partner in crime for AF treatment since the PARP protein plays an important role in the repair of ROS-induced DNA damage. Previously, olaparib has been combined with TrxR inhibitors APR-246 (PRIMA-1^MET^) or alantolactone, which resulted in synergistic cytotoxicity in cancer cells. However, these compounds are still in preclinical or early-stage clinical development. Therefore we hypothesized that similar synergistic effects could be obtained using a repurposed FDA-approved drug in combination with olaparib to limit the cost and time to translate this combination strategy to the patient [22,25,26,48]. In this study, we demonstrate that the response to AF is synergistically enhanced by olaparib treatment in mutant p53 NSCLC and PDAC cell cultures in vitro. These results were confirmed in vivo since simultaneous delivery of AF and olaparib via oral gavage significantly reduced tumor kinetics compared to monotherapies.

Cancer cells lacking functional BRCA1 or BRCA2, critical players in homologous recombination repair, are more sensitive to PARP inhibition [49]. Consequently, olaparib is mainly investigated in the context of BRCA-deficient cancer types and has already been approved by the European Medicines Agency (EMA) and US FDA for the treatment of recurrent ovarian cancer in *BRCA1/2*-mutated women [50]. This restricts the clinical benefit of olaparib to only a small percentage of patients who carry mutant *BRCA* genes [51]. Next to *BRCA* mutation, the most frequent somatic genetic event in ovarian cancer and many other cancer types is a mutation of the *TP53* gene [52]. Inactivating mutations in the *TP53* gene can turn mutant p53 from a strength for the tumor into a weakness by rendering mutant p53 tumors more susceptible to oxidative damage [7,8]. We show that higher levels of mutant p53 protein increased the cytotoxic response to olaparib. Interestingly, we observed that therapy-induced senescence was selectively induced in the p53 WT A549 cells but not in their mutant p53 counterpart, further proving the critical role of p53 in the response to olaparib treatment. Similarly, a study by Liu et al. showed that olaparib treatment was preferentially cytotoxic to *TP53*-mutant but not WT cell lines derived from muscle-invasive bladder cancer and NSCLC [27], and Wang et al. showed that low concentrations (5 μM) of olaparib induced cellular senescence through the p16/p53 pathway in both WT and mutant p53 ovarian cancer cells using senescence-associated β-galactosidase staining [53]. In addition, our previous data have already identified the presence of mutant p53 overexpression as an important sensitizer for AF treatment in (isogenic) NSCLC cells (14). This sensitizing effect of mutant p53 was retained in the aurola combination strategy since mutant p53 A549.R2 cells were much more sensitive to aurola treatment compared to p53 WT A549 cells.

Our research group has already demonstrated that the reactivator of mutant p53 APR-246 enhanced the cytotoxic response of olaparib in mutant p53 NSCLC cells, resulting in a strong induction of apoptosis [25]. Despite some promising results, APR-246 is still the only mutant p53-reactivating molecule that reached an advanced stage of clinical investigation with a phase III clinical trial in *TP53* mutant myelodysplastic syndrome (NCT03745716) [54]. Next to its p53-dependent activities, APR-246 also targets the cellular redox balance by inhibiting TrxR, leading to the induction of ROS [48]. Therefore, the TrxR inhibitor AF is a promising alternative treatment in combination with olaparib since it has similar features as APR-246 but with better and faster perspectives to reach the clinic as an FDA-approved drug. Here, we showed that the combination of AF and olaparib synergistically enhanced the induction of ROS-dependent cell death in 2D cultures of mutant p53 NSCLC and PDAC cells, thereby confirming our hypothesis. Similarly, other natural compounds that function as TrxR inhibitors, such as alantolactone or alkannin, markedly synergized with olaparib to yield a synergistic cytotoxic response in both prostate and colorectal cancer in vitro and regression of tumor xenografts in vivo [22,55]. These results highlight the potential of combining oxidative stress inducers and PARP inhibition both in vitro and in vivo as broad-spectrum anticancer therapy.

Compared to normal cells, the production of ROS is enhanced in tumor cells due to an increased metabolic rate, gene mutation, and hypoxia in the tumor microenvironment (TME) [56]. The role of ROS was investigated to elucidate the underlying mechanisms of aurola-mediated cell death. We demonstrated that ROS significantly accumulated in all mutant p53 cell lines after treatment with aurola. We confirmed this ROS-mediated response in vitro, as the ROS scavenger NAC protected all cell lines from aurola-mediated cell death, supporting the driving influence of ROS. Cellular ROS levels are strictly controlled by balancing ROS generating and scavenging systems, which includes the redox protein thioredoxin (Trx). It is already well established that AF-mediated TrxR inhibition modulates the intracellular redox state and increases ROS levels in different cancer types [14,18,57,58]. Olaparib induced a significant increase in ROS levels only in mutant p53 NSCLC NCI-H2228 cells. Consequently, NAC slightly decreased olaparib-induced cell death in these NCI-H2228 cells but did not reverse olaparib-induced cell death in the three other cell lines. However, it has been reported that PARP-1 has been implicated in the regulation of mitochondrial content, oxidative metabolism, and ROS production [59]. To this end, PARP-1 can also modulate the transcription of the redox regulator NRF-2 [60]. Other studies proposed that PARP inhibition increases ROS by upregulating nicotinamide adenine dinucleotide phosphate (NADPH) oxidase (NOX) [29]. A study by Ma et al. reported that a lethal dose of 2 to 10 μM olaparib resulted in a significant increase in ROS production in breast cancer cells in vitro [61]. In bladder cell lines, olaparib cytotoxicity was mediated by elevated mitochondrial ROS levels both in vitro and ex vivo in tissue explants [27].

These elevated ROS levels can induce oxidative DNA damage and regulate different types of cancer cell death, including apoptosis but also ferroptosis [45,62]. Oxidative DNA damage and ROS induction have already been shown to synergistically induce cancer lethality with PARP inhibitors [22,25,27,28,29,63,64]. To date, most studies have shown that PARP inhibitors such as rucaparib, PJ34, and olaparib promote apoptosis in different cancer types [23,65,66]. However, it has been reported that olaparib could suppress tumors through mechanisms that are not directly linked to DNA damage and apoptosis [67,68]. We are the first to show that the aurola combination treatment sensitized mutant p53 NSCLC cells for caspase-3/7-dependent apoptosis, as well as for lipid peroxidation-dependent ferroptosis. Only in the apoptotic NCI-H2228 cells treated with the combination of AF and olaparib did the increasing trend in γH2AX spots indicate more double-stranded breaks and relative DNA damage. This suggests that aurola-mediated apoptotic cell death is potentially driven by an increase in DNA damage. However, in A549.R2 cells, which are more prone to ferroptosis, aurola treatment did not result in more double-stranded DNA breaks compared to olaparib monotherapy, supporting the alternative cell death mechanism through lipid peroxidation. Ferroptosis depends on the presence of intracellular iron and the induction of ROS [56]. The aurola-mediated increase in ROS could explain the induction of ferroptosis. Recently, we already demonstrated the induction of ferroptosis and apoptosis in isogenic mutant p53 NSCLC cells after AF treatment [14]. A study by Hong et al. showed that PARP inhibition promoted ferroptosis by repressing SLC7A11-mediated glutathione (GSH) biosynthesis in a p53-dependent manner and synergized with ferroptosis inducers in BRCA-proficient ovarian cancer [67].

Murine adenocarcinoma lung cancer cell line 344SQ, derived from KrasLa1/+p53R172HΔG mice, was, due to its genetic background, a relevant in vivo model to study the combination of AF and olaparib in a mutant p53 setting. To first validate aurola in these murine cells in vitro, a targeted titration series of AF and olaparib was performed around the 3 µM clinically relevant reference point of olaparib in 2D and 3D 344SQ cultures to avoid high concentrations of olaparib. However, only in 3D 344SQ spheroid cultures was a synergistic interaction observed between the highest concentration of AF and olaparib. Similar to the mutant p53 NSCLC and PDAC cells used in this study, we expect that higher concentrations of olaparib are required to induce synergistic treatment responses in combination with AF in both 2D and 3D 344SQ cultures. Overall, these results emphasize that relatively high concentrations of AF and olaparib are necessary to induce a synergistic effect in vitro. As a result, the choice of concentration for in vitro or in vivo testing of a drug can be a challenge in cancer research. High concentrations used in vitro are often required in the cell culture medium to induce cell damage compared to plasma peak concentrations that are known to cause adverse effects in vivo. [69]. Importantly, it might be challenging to obtain sufficiently high concentrations of AF and olaparib in a patient’s tumor without increasing unwanted side effects. New technological innovations such as AF-loaded nanoparticles could offer a possible solution since they are able to enhance drug localization in the tumor and minimize systemic toxicity [70,71].

Despite the high in vitro concentration range required for synergy, the simultaneous combination regiment of AF and olaparib at tolerable concentrations for 14 days induced a delay in tumor growth compared to the monotherapies and an increase in survival compared to vehicle in a syngeneic lung adenocarcinoma 344SQ tumor-bearing 129 mouse model. The combination was well tolerated without weight loss in the mice. Both compounds were given orally to mice via oral gavage, which mimics the clinical administration of both AF and olaparib as capsules to RA patients and cancer patients, respectively. Since AF was the first orally active gold compound for the treatment of RA patients, several in vitro and in vivo studies before 1990 tried to unravel its mechanisms of action against RA and investigate the effects of AF on cell-mediated immunity [72]. RNAseq data showed that AF induced the down-regulation of immune-related pathways after 14 days of treatment in the 344SQ mice tumors, which was also seen in the aurola combination treatment. This is in accordance with AF’s anti-inflammatory function, as it has been shown that AF manages the autoimmune response via the inhibition of immune cell infiltration to the site of inflammation via T cell mitogenesis and macrophage cytotoxicity [58]. AF also indirectly reduces the secretion of pro-inflammatory cytokines (IL-6 and IL-8) from macrophages and monocytes via the inhibition of the NF-kB signaling pathway [73,74]. Nevertheless, AF has been shown to enhance the efficacy of PD-L1 blockage in triple-negative breast cancer mouse models, indicating that this anti-inflammatory response might be valuable in combination with immunotherapy [75]. In contrast to AF, olaparib has a more beneficial effect on immune-related pathways in vivo. Multiple studies already reported on the beneficial effects of PARP inhibitors on the immune system in vivo. Olaparib treatment was associated with an activated effector T-cell response and reduction of immune-checkpoint receptor expression in in vivo models of BRCA1-deficient ovarian cancer [76]. In addition, olaparib treatment drove immune cell infiltration and activation, and the expression of immune STING/Type I IFN pathways in a BRCA mouse model [77]. Next to immune related pathway, we also checked the effect of AF, olaparib and the combination on cell cycle, apoptosis and DNA repair pathways. Only tumors of mice treated with the aurola combination showed significant downregulation of cell cycle genes. If this effect could explain the inhibitory effect of the combination on tumor kinetics should be investigated further in more depth. In literature, quantitative proteomic comparison of AF-treated breast cancer cells revealed that cell proliferation, cell division and cell cycle were among the most affected pathways [78]. Since tumor xenografts are the most widely used model to test AF treatment in vivo [40], we were the first to investigate aurola in this syngeneic 344SQ tumor-bearing 129 mouse model. However, a limitation of the present study is the use of s.c.-injected tumor cells that does not completely mimic the human situation. Therefore, an orthotopic lung model that creates a more disease-relevant environment in the lung would be beneficial for future research. Additionally, it would also have been beneficial to test other treatment regimens, such as a sequential combination of first blocking PARP-mediated DNA repair via olaparib and second further accumulating ROS-mediated DNA damage via AF.

Overall, we confirmed our hypothesis that AF-mediated oxidative stress induction in combination with olaparib-mediated PARP inhibition could induce a cytotoxic and synergistic response against mutant p53 cancers in vitro and a reduction of tumor growth in vivo. This highlights the therapeutic value of combining AF with olaparib in mutant p53 cancer patients in which resistance to standard therapies often occurs.

## 5. Conclusions

We demonstrated the effectiveness of the aurola combination regimen to synergistically eradicate NSCLC and PDAC cells in vitro via different ROS-dependent cell death mechanisms, namely apoptosis and ferroptosis. In a mutant p53 syngeneic lung adenocarcinoma mouse model, simultaneous treatment of AF and olaparib significantly delayed tumor growth compared to monotherapies. Thus, this study supports the use of the synergistic cytotoxicity between pro-oxidative agents and PARP inhibitors to exploit cancer vulnerabilities common to most tumor cells. Since the combination attacks two very important vulnerabilities of cancer cells, specifically higher ROS levels and impaired DNA damage response, it can induce an efficient elimination of tumors cells with a lower chance of resistance. In conclusion, our findings suggest a promising combination strategy for mutant p53 cancers in which the response to AF is synergistically enhanced by the addition of olaparib, although high concentrations of both compounds need to be reached to obtain a substantial cytotoxic effect.

## Data Availability

The data are contained within the article and Supplementary Materials.

## Supplementary Materials

The following supporting information can be downloaded at: https://www.mdpi.com/article/10.3390/antiox12030667/s1, Table S1: P53 status of NSCLC and PDAC cell lines; Figure S1: Baseline expression of p53 protein and its effect on AF and olaparib treatment response in NSCLC and PDAC cell lines; Figure S2: Expression of p53 protein in NSCLC knock-down cell panel of p53; Figure S3: Synergistic interaction between AF and olaparib in NSCLC and PDAC cells in vitro; Figure S4: Effect of p53 status on cytotoxic response of AF and olaparib; Figure S5: The effect of the ROS scavenger NAC on aurola-mediated cytotoxicity in NSCLC and PDAC cell lines in vitro; Figure S6: Induction of cellular senescence after treatment with olaparib in WT p53 A549 cells; Figure S7: Double-stranded break detection in mutant p53 NSCLC cell lines NCI-H2228 and A549.R2; Figure S8: Effect of AF, olaparib, and aurola treatment on body weight of 129S2/SvPasCrl (129) mice; Figure S9: Clustering profile of AF, olaparib, and aurola-treated 344SQ tumors; Figure S10: Hierarchical clustering of AF, olaparib, and aurola-treated mice tumors on gene level.

## References

[1] Siegel R.L., Miller K.D., Fuchs H.E., Jemal A. (2021). Cancer Statistics, 2021. CA Cancer J. Clin..

[2] Uzunparmak B., Sahin I.H. (2019). Pancreatic cancer microenvironment: A current dilemma. Clin. Transl. Med..

[3] Gibbons D.L., Byers L.A., Kurie J.M. (2014). Smoking, p53 mutation, and lung cancer. Mol. Cancer Res..

[4] Deben C., Bossche J.V.D., Van Der Steen N., Lardon F., Wouters A., de Beeck K.O., Hermans C., Jacobs J., Peeters M., Van Camp G. (2017). Deep sequencing of the *TP53* gene reveals a potential risk allele for non–small cell lung cancer and supports the negative prognostic value of *TP53* variants. Tumor Biol..

[5] Greulich H. (2010). The Genomics of Lung Adenocarcinoma: Opportunities for Targeted Therapies. Genes Cancer.

[6] Cicenas J., Kvederaviciute K., Meskinyte I., Meskinyte-Kausiliene E., Skeberdyte A., Cicenas J. (2017). KRAS, TP53, CDKN2A, SMAD4, BRCA1, and BRCA2 Mutations in Pancreatic Cancer. Cancers.

[7] Liu D.S., Duong C.P., Haupt S., Montgomery K.G., House C.M., Azar W.J., Pearson H.B., Fisher O.M., Read M., Guerra G.R. (2017). Inhibiting the system xC−/glutathione axis selectively targets cancers with mutant-p53 accumulation. Nat. Commun..

[8] Kalo E., Kogan-Sakin I., Solomon H., Bar-Nathan E., Shay M., Shetzer Y., Dekel E., Goldfinger N., Buganim Y., Stambolsky P. (2012). Mutant p53R273H attenuates the expression of phase 2 detoxifying enzymes and promotes the survival of cells with high levels of reactive oxygen species. J. Cell Sci..

[9] Trachootham D., Alexandre J., Huang P. (2009). Targeting cancer cells by ROS-mediated mechanisms: A radical therapeutic approach?. Nat. Rev. Drug. Discov..

[10] Liu J., Wang Z. (2015). Increased Oxidative Stress as a Selective Anticancer Therapy. Oxidative Med. Cell. Longev..

[11] Nogueira V., Hay N. (2013). Molecular Pathways: Reactive Oxygen Species Homeostasis in Cancer Cells and Implications for Cancer Therapy. Clin. Cancer Res..

[12] Roder C., Thomson M.J. (2015). Auranofin: Repurposing an Old Drug for a Golden New Age. Drugs R&D.

[13] Pantziarka P., Verbaanderd C., Sukhatme V., Capistrano I.R., Crispino S., Gyawali B., Rooman I., Van Nuffel A., Meheus L., Sukhatme V.P. (2018). ReDO_DB: The repurposing drugs in oncology database. Ecancermedicalscience.

[14] Boullosa L.F., Van Loenhout J., Flieswasser T., De Waele J., Hermans C., Lambrechts H., Cuypers B., Laukens K., Bartholomeus E., Siozopoulou V. (2021). Auranofin reveals therapeutic anticancer potential by triggering distinct molecular cell death mechanisms and innate immunity in mutant p53 non-small cell lung cancer. Redox Biol..

[15] DeBerardinis R.J., Chandel N.S. (2016). Fundamentals of cancer metabolism. Sci. Adv..

[16] Hwang-Bo H., Jeong J.-W., Han M.H., Park C., Hong S.-H., Kim G.-Y., Moon S.-K., Cheong J., Kim W.-J., Yoo Y.H. (2017). Auranofin, an inhibitor of thioredoxin reductase, induces apoptosis in hepatocellular carcinoma Hep3B cells by generation of reactive oxygen species. Gen. Physiol. Biophys..

[17] Fiskus W., Saba N., Shen M., Ghias M., Liu J., Das Gupta S., Chauhan L., Rao R., Gunewardena S., Schorno K. (2014). Auranofin Induces Lethal Oxidative and Endoplasmic Reticulum Stress and Exerts Potent Preclinical Activity against Chronic Lymphocytic Leukemia. Cancer Res..

[18] Zou P., Chen M., Ji J., Chen W., Chen X., Ying S., Zhang J., Zhang Z., Liu Z., Yang S. (2015). Auranofin induces apoptosis by ROS-mediated ER stress and mitochondrial dysfunction and displayed synergistic lethality with piperlongumine in gastric cancer. Oncotarget.

[19] Kim I.S., Jin J.Y., Lee I.H., Park S.J. (2004). Auranofin induces apoptosis and when combined with retinoic acid enhances differentiation of acute promyelocytic leukaemia cells in vitro. Br. J. Pharmacol..

[20] Karsa M., Kosciolek A., Bongers A., Mariana A., Failes T., Gifford A.J., Kees U.R., Cheung L.C., Kotecha R.S., Arndt G.M. (2021). Exploiting the reactive oxygen species imbalance in high-risk paediatric acute lymphoblastic leukaemia through auranofin. Br. J. Cancer.

[21] Li H., Hu J., Wu S., Wang L., Cao X., Zhang X., Dai B., Cao M., Shao R., Zhang R. (2015). Auranofin-mediated inhibition of PI3K/AKT/mTOR axis and anticancer activity in non-small cell lung cancer cells. Oncotarget.

[22] Wang H., Zhang S., Song L., Qu M., Zou Z. (2020). Synergistic lethality between PARP-trapping and alantolactone-induced oxidative DNA damage in homologous recombination-proficient cancer cells. Oncogene.

[23] Kim D., Nam H.J. (2022). PARP Inhibitors: Clinical Limitations and Recent Attempts to Overcome Them. Int. J. Mol. Sci..

[24] Farmer H., McCabe N., Lord C.J., Tutt A.N.J., Johnson D.A., Richardson T.B., Santarosa M., Dillon K.J., Hickson I., Knights C. (2005). Targeting the DNA repair defect in BRCA mutant cells as a therapeutic strategy. Nature.

[25] Deben C., Lardon F., Wouters A., de Beeck K.O., Van den Bossche J., Jacobs J., Van Der Steen N., Peeters M., Rolfo C., Deschoolmeester V. (2016). APR-246 (PRIMA-1 MET) strongly synergizes with AZD2281 (olaparib) induced PARP inhibition to induce apoptosis in non-small cell lung cancer cell lines. Cancer Lett..

[26] Yin Z.-X., Hang W., Liu G., Wang Y.-S., Shen X.-F., Sun Q.-H., Li D.-D., Jian Y.-P., Zhang Y.-H., Quan C.-S. (2017). PARP-1 inhibitors sensitize HNSCC cells to APR-246 by inactivation of thioredoxin reductase 1 (TrxR1) and promotion of ROS accumulation. Oncotarget.

[27] Liu Q., Gheorghiu L., Drumm M., Clayman R., Eidelman A., Wszolek M.F., Olumi A., Feldman A., Wang M., Marcar L. (2018). PARP-1 inhibition with or without ionizing radiation confers reactive oxygen species-mediated cytotoxicity preferentially to cancer cells with mutant TP53. Oncogene.

[28] Huang X., Motea E.A., Moore Z.R., Yao J., Dong Y., Chakrabarti G., Kilgore J.A., Silvers M.A., Patidar P.L., Cholka A. (2016). Leveraging an NQO1 Bioactivatable Drug for Tumor-Selective Use of Poly(ADP-ribose) Polymerase Inhibitors. Cancer Cell.

[29] Marcar L., Bardhan K., Gheorghiu L., Dinkelborg P., Pfäffle H., Liu Q., Wang M., Piotrowska Z., Sequist L.V., Borgmann K. (2019). Acquired Resistance of EGFR-Mutated Lung Cancer to Tyrosine Kinase Inhibitor Treatment Promotes PARP Inhibitor Sensitivity. Cell Rep..

[30] Blandino G., Levine A.J., Oren M. (1999). Mutant p53 gain of function: Differential effects of different p53 mutants on resistance of cultured cells to chemotherapy. Oncogene.

[31] Deben C., Deschoolmeester V., De Waele J., Jacobs J., Bossche J.V.D., Wouters A., Peeters M., Rolfo C., Smits E., Lardon F. (2018). Hypoxia-Induced Cisplatin Resistance in Non-Small Cell Lung Cancer Cells Is Mediated by HIF-1α and Mutant p53 and Can Be Overcome by Induction of Oxidative Stress. Cancers.

[32] Deben C., Boullosa L.F., Domen A., Wouters A., Cuypers B., Laukens K., Lardon F., Pauwels P. (2021). Characterization of acquired nutlin-3 resistant non-small cell lung cancer cells. Cancer Drug Resist..

[33] Pauwels B., Korst A.E.C., De Pooter C.M.J., Pattyn G.G.O., Lambrechts H.A.J., Baay M.F.D., Lardon F., Vermorken J.B. (2003). Comparison of the sulforhodamine B assay and the clonogenic assay for in vitro chemoradiation studies. Cancer Chemother. Pharmacol..

[34] Ianevski A., Giri A.K., Aittokallio T. (2022). SynergyFinder 3.0: An interactive analysis and consensus interpretation of multi-drug synergies across multiple samples. Nucleic Acids Res..

[35] Fiji. https://fiji.sc.

[36] De Vos W.H., Van Neste L., Dieriks B., Joss G.H., Van Oostveldt P. (2010). High content image cytometry in the context of subnuclear organization. Cytom. A.

[37] Schmidt U., Weigert M., Broaddus C., Myers G. (2018). Cell Detection with Star-Convex Polygons. Proceedings of the International Conference on Medical Image Computing and Computer-Assisted Intervention.

[38] Deben C., De La Hoz E.C., Le Compte M., Van Schil P., Hendriks J.M., Lauwers P., Yogeswaran S.K., Lardon F., Pauwels P., Van Laere S. (2022). OrBITS: Label-free and time-lapse monitoring of patient derived organoids for advanced drug screening. Cell. Oncol..

[39] Gupta A., Gautam P., Wennerberg K., Aittokallio T. (2020). A normalized drug response metric improves accuracy and consistency of anticancer drug sensitivity quantification in cell-based screening. Commun. Biol..

[40] Freire Boullosa L., Van Loenhout J., Hermans C., Lau H.W., Merlin C., Marcq E., Takhsha F.S., Martinet W., De Meyer G.R., Lardon F. (2022). Optimization of the Solvent and In Vivo Administration Route of Auranofin in a Syngeneic Non-Small Cell Lung Cancer and Glioblastoma Mouse Model. Pharmaceutics.

[41] Akhmedov M., Martinelli A., Geiger R., Kwee I. (2019). Omics Playground: A comprehensive self-service platform for visualization, analytics and exploration of Big Omics Data. NAR Genom. Bioinform..

[42] R Foundation for Statistical Computing (2018). R: A Language and Environment for Statistical Computing.

[43] Fisher S.A., Peddle-McIntyre C.J., Burton K., Newton R.U., Marcq E., Lake R.A., Nowak A.K. (2020). Voluntary exercise in mesothelioma: Effects on tumour growth and treatment response in a murine model. BMC Res. Notes.

[44] AbdulSalam S.F., Thowfeik F.S., Merino E.J. (2016). Excessive Reactive Oxygen Species and Exotic DNA Lesions as an Exploitable Liability. Biochemistry.

[45] Gorrini C., Harris I.S., Mak T.W. (2013). Modulation of oxidative stress as an anticancer strategy. Nat. Rev. Drug Discov..

[46] Mou Y., Wang J., Wu J., He D., Zhang C., Duan C., Li B. (2019). Ferroptosis, a new form of cell death: Opportunities and challenges in cancer. J. Hematol. Oncol..

[47] Schieber M., Chandel N.S. (2014). ROS Function in Redox Signaling and Oxidative Stress. Curr. Biol..

[48] Peng X., Zhang M.Q.Z., Conserva F., Hosny G., Selivanova G., Bykov V.J.N., Arnér E.S.J., Wiman K.G. (2013). APR-246/PRIMA-1MET inhibits thioredoxin reductase 1 and converts the enzyme to a dedicated NADPH oxidase. Cell Death Dis..

[49] Li H., Liu Z.-Y., Wu N., Chen Y.-C., Cheng Q., Wang J. (2020). PARP inhibitor resistance: The underlying mechanisms and clinical implications. Mol. Cancer.

[50] Kim G., Ison G., McKee A.E., Zhang H., Tang S., Gwise T., Sridhara R., Lee E., Tzou A., Philip R. (2015). FDA Approval Summary: Olaparib Monotherapy in Patients with Deleterious Germline BRCA-Mutated Advanced Ovarian Cancer Treated with Three or More Lines of Chemotherapy. Clin. Cancer Res..

[51] Alsop K., Fereday S., Meldrum C., DeFazio A., Emmanuel C., George J., Dobrovic A., Birrer M.J., Webb P.M., Stewart C. (2012). BRCA Mutation Frequency and Patterns of Treatment Response in BRCA Mutation–Positive Women with Ovarian Cancer: A Report from the Australian Ovarian Cancer Study Group. J. Clin. Oncol..

[52] Zweemer R.P., Shaw P.A., Verheijen R.M., Ryan A., Berchuck A., Ponder B.A., Risch H., McLaughlin J.R., Narod S.A., Menko F.H. (1999). Accumulation of p53 protein is frequent in ovarian cancers associated with BRCA1 and BRCA2 germline mutations. J. Clin. Pathol..

[53] Wang Z., Gao J., Zhou J., Liu H., Xu C. (2019). Olaparib induced senescence under P16 or P53 dependent manner in ovarian cancer. J. Gynecol. Oncol..

[54] US National Library of Medicine. https://clinicaltrials.gov.

[55] Chang M., Wang H., Niu J., Song Y., Zou Z. (2020). Alkannin-Induced Oxidative DNA Damage Synergizes with PARP Inhibition to Cause Cancer-Specific Cytotoxicity. Front. Pharmacol..

[56] Perillo B., Di Donato M., Pezone A., Di Zazzo E., Giovannelli P., Galasso G., Castoria G., Migliaccio A. (2020). ROS in cancer therapy: The bright side of the moon. Exp. Mol. Med..

[57] Van Loenhout J., Freire Boullosa L., Quatannens D., De Waele J., Merlin C., Lambrechts H., Lau H.W., Hermans C., Lin A., Lardon F. (2021). Auranofin and Cold Atmospheric Plasma Synergize to Trigger Distinct Cell Death Mechanisms and Immunogenic Responses in Glioblastoma. Cells.

[58] Abdalbari F.H., Telleria C.M. (2021). The gold complex auranofin: New perspectives for cancer therapy. Discov. Oncol..

[59] Bai P., Cantó C., Oudart H., Brunyánszki A., Cen Y., Thomas C., Yamamoto H., Huber A., Kiss B., Houtkooper R.H. (2011). PARP-1 Inhibition Increases Mitochondrial Metabolism through SIRT1 Activation. Cell Metab..

[60] Wu T., Wang X.-J., Tian W., Jaramillo M.C., Lau A., Zhang D.D. (2014). Poly(ADP-ribose) polymerase-1 modulates Nrf2-dependent transcription. Free Radic. Biol. Med..

[61] Ma X., Dang C., Min W., Diao Y., Hui W., Wang X., Dai Z., Wang X., Kang H. (2019). Downregulation of APE1 potentiates breast cancer cells to olaparib by inhibiting PARP-1 expression. Breast Cancer Res. Treat..

[62] Villalpando-Rodriguez G.E., Gibson S.B. (2021). Reactive Oxygen Species (ROS) Regulates Different Types of Cell Death by Acting as a Rheostat. Oxidative Med. Cell. Longev..

[63] Pulliam N., Fang F., Ozes A.R., Tang J., Adewuyi A., Keer H., Lyons J., Baylin S.B., Matei D., Nakshatri H. (2018). An Effective Epigenetic-PARP Inhibitor Combination Therapy for Breast and Ovarian Cancers Independent of BRCA Mutations. Clin. Cancer Res..

[64] Gralewska P., Gajek A., Marczak A., Rogalska A. (2021). Metformin Affects Olaparib Sensitivity through Induction of Apoptosis in Epithelial Ovarian Cancer Cell Lines. Int. J. Mol. Sci..

[65] Gaymes T.J., Shall S., MacPherson L.J., Twine N.A., Lea N.C., Farzaneh F., Mufti G.J. (2009). Inhibitors of poly ADP-ribose polymerase (PARP) induce apoptosis of myeloid leukemic cells: Potential for therapy of myeloid leukemia and myelodysplastic syndromes. Haematologica.

[66] Shi Y., Zhou F., Jiang F., Lu H., Wang J., Cheng C. (2014). PARP inhibitor reduces proliferation and increases apoptosis in breast cancer cells. Chin. J. Cancer Res..

[67] Hong T., Lei G., Chen X., Li H., Zhang X., Wu N., Zhao Y., Zhang Y., Wang J. (2021). PARP inhibition promotes ferroptosis via repressing SLC7A11 and synergizes with ferroptosis inducers in BRCA-proficient ovarian cancer. Redox Biol..

[68] Macciò A., Madeddu C. (2019). The mechanism of cancer cell death by PARP inhibitors goes beyond DNA damage alone. Int. J. Cancer.

[69] Albrecht W. (2020). Which concentrations are optimal for in vitro testing?. EXCLI J..

[70] Díez-Martínez R., García-Fernández E., Manzano M., Martínez Á., Domenech M., Vallet-Regí M., García P. (2016). Auranofin-loaded nanoparticles as a new therapeutic tool to fight streptococcal infections. Sci. Rep..

[71] Awasthi R., Roseblade A., Hansbro P., Rathbone M.J., Dua K., Bebawy M. (2018). Nanoparticles in Cancer Treatment: Opportunities and Obstacles. Curr. Drug Targets.

[72] Chaffman M., Brogden R.N., Heel R.C., Speight T.M., Avery G.S. (1984). Auranofin. A preliminary review of its pharmacological properties and therapeutic use in rheumatoid arthritis. Drugs.

[73] Kim N.-H., Lee M.-Y., Park S.-J., Choi J.-S., Oh M.-K., Kim I.-S. (2007). Auranofin blocks interleukin-6 signalling by inhibiting phosphorylation of JAK1 and STAT3. Immunology.

[74] Jeon K.-I., Jeong J.-Y., Jue D.-M. (2000). Thiol-Reactive Metal Compounds Inhibit NF-κB Activation by Blocking IκB Kinase. J. Immunol..

[75] Raninga P.V., Lee A.C., Sinha D., Shih Y.Y., Mittal D., Makhale A., Bain A.L., Nanayakarra D., Tonissen K.F., Kalimutho M. (2020). Therapeutic cooperation between auranofin, a thioredoxin reductase inhibitor and anti-PD-L1 antibody for treatment of triple-negative breast cancer. Int. J. Cancer.

[76] Lee E.K., Konstantinopoulos P.A. (2020). PARP inhibition and immune modulation: Scientific rationale and perspectives for the treatment of gynecologic cancers. Ther. Adv. Med. Oncol..

[77] Staniszewska A.D., Armenia J., King M., Michaloglou C., Reddy A., Singh M., Martin M.S., Prickett L., Wilson Z., Proia T. (2022). PARP inhibition is a modulator of anti-tumor immune response in BRCA-deficient tumors. Oncoimmunology.

[78] Hatem E., El Banna N., Heneman-Masurel A., Baïlle D., Vernis L., Riquier S., Golinelli-Cohen M.-P., Guittet O., Vallières C., Camadro J.-M. (2022). Novel Insights into Redox-Based Mechanisms for Auranofin-Induced Rapid Cancer Cell Death. Cancers.

