# Supplementary material for: Auranofin Synergizes with the PARP Inhibitor Olaparib to Induce ROS-Mediated Cell Death in Mutant p53 Cancers
Source: Antioxidants (Basel). 2023 Mar 8;12(3):667. doi: 10.3390/antiox12030667 (PMC10045521; doi:10.3390/antiox12030667)

SUPPLEMENTARY FILE

1. SUPPLEMENTARY METHODS

Analysis of cellular senescence

Olaparib-treated A549 and A549.R2 cells were fixed at room temperature with 2 % paraformaldehyde (PFA) and stained at pH 6.0 using a senescence β-galactosidase staining kit (Cell Signaling Technology). Plates were incubated with X-gal staining solution overnight at 37°C in a dry incubator without CO_2. Using a transmitted-light microscope (Olympus BX41), equipped with a Leica DFC450C camera, blue staining was visualized.

2. SUPPLEMENTARY TABLES

Table S1. P53 status of NSCLC and PDAC cell lines

Cell line	Origin	Protein phenotype	Functional phenotype
Mia-PaCa-2	PDAC	p.R248W	Missense
Panc-1	PDAC	p.R273H	Missense
Capan-2	PDAC	p.T125=	Silencing
BxPC-3	PDAC	p.Y220C	Missense
NCI-H1975	NSCLC	p.R273H	Missense
NCI-H2228	NSCLC	p.Q331*	Nonsense
NCI-H2228 – NTC	NSCLC	p.Q331*	Nonsense
NCI-H2228 – *TP53* shRNA	NSCLC	p.Q331*	Knock-down
NCI-H596	NSCLC	p.G245C	Missense
A549	NSCLC	WT	WT
A549.R2	NSCLC	p.Y236N/R248W	Missense
NCI-H1299	NSCLC	Null	Null
NCI-H1299 – R175H	NSCLC	p.R175H	Missense
NCI-H1299 – R273H	NSCLC	p.R273H	Missense

PDAC: pancreatic ductal adenocarcinoma; NSCLC: non-small cell lung cancer; WT: wild-type. NTC: non-template control; shRNA: short hairpin RNA; *: truncated protein due to nonsense mutation.

3. SUPPLEMENTARY FIGURES

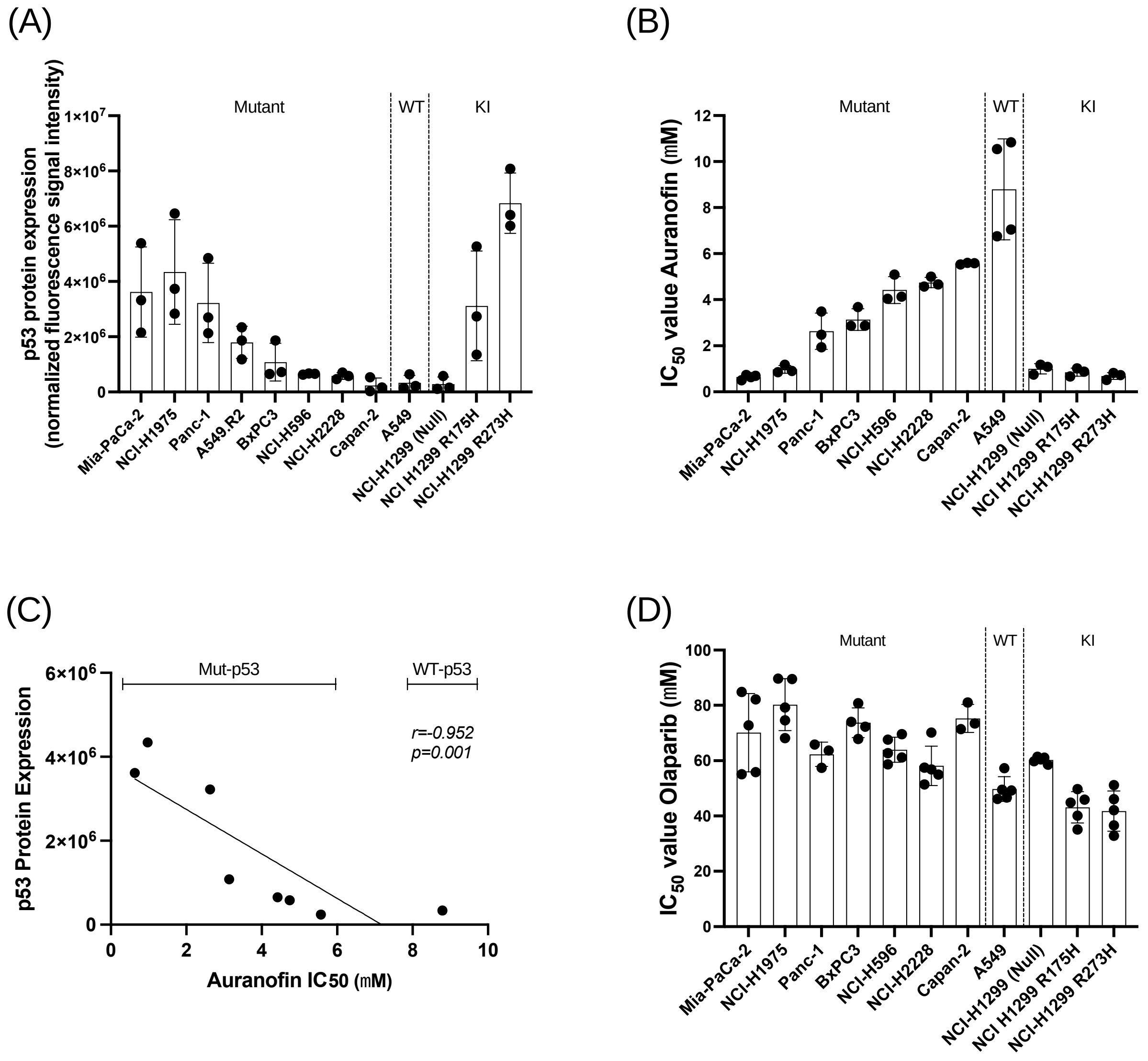

Figure S1. Baseline expression of p53 protein and its effect on AF and olaparib treatment response in NSCLC and PDAC cell lines. (**A**) Baseline p53 protein expression (determined by western blotting) in a panel of 12 (isogenic) NSCLC and PDAC cell lines with different p53 backgrounds. (**B**) IC_{50} values (µM) after 72 h of AF treatment in a panel of 11 (isogenic) NSCLC and PDAC cell lines with different p53 backgrounds (determined by SRB assay). (**C**) Spearman's r correlation between baseline p53 protein expression (determined by western blotting) and AF IC_{50} values (µM) in a panel of 8 NSCLC and PDAC cell lines with different p53 backgrounds. (**D**) IC_{50} values (µM) after 72 h of olaparib treatment in a panel of 11 (isogenic) NSCLC and PDAC cell lines with different p53 backgrounds (determined by SRB assay). WT: Wild-type p53; KI: knock-in p53. Experiments were performed at least in triplicate. Error bars represent the standard deviation. $^{*}p \leq 0.05$ significant differences.

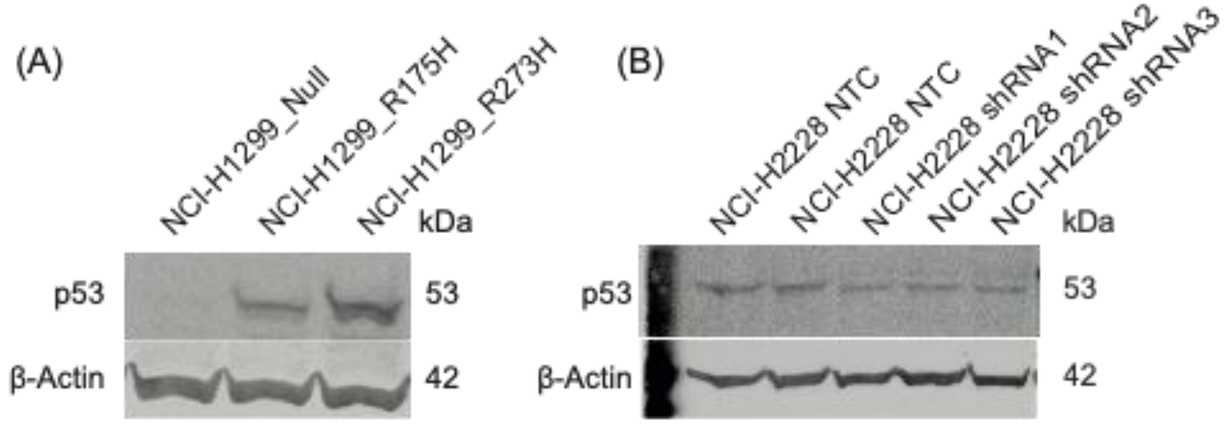

Figure S2. Expression of p53 protein in NSCLC knock-in and knock-down cell panel of p53. Western blots representing the baseline protein expression of p53 in the isogenic NCI-H1299 NSCLC cell lines (**A**) and in the non-template control (NTC) NSCLC cell line NCI-H2228 and NCI-H2228 cells with knockdown of *TP53* via shRNA1-3 (**B**). β-actin was used as internal control. Length of the proteins are represented in kilodalton (kDa).

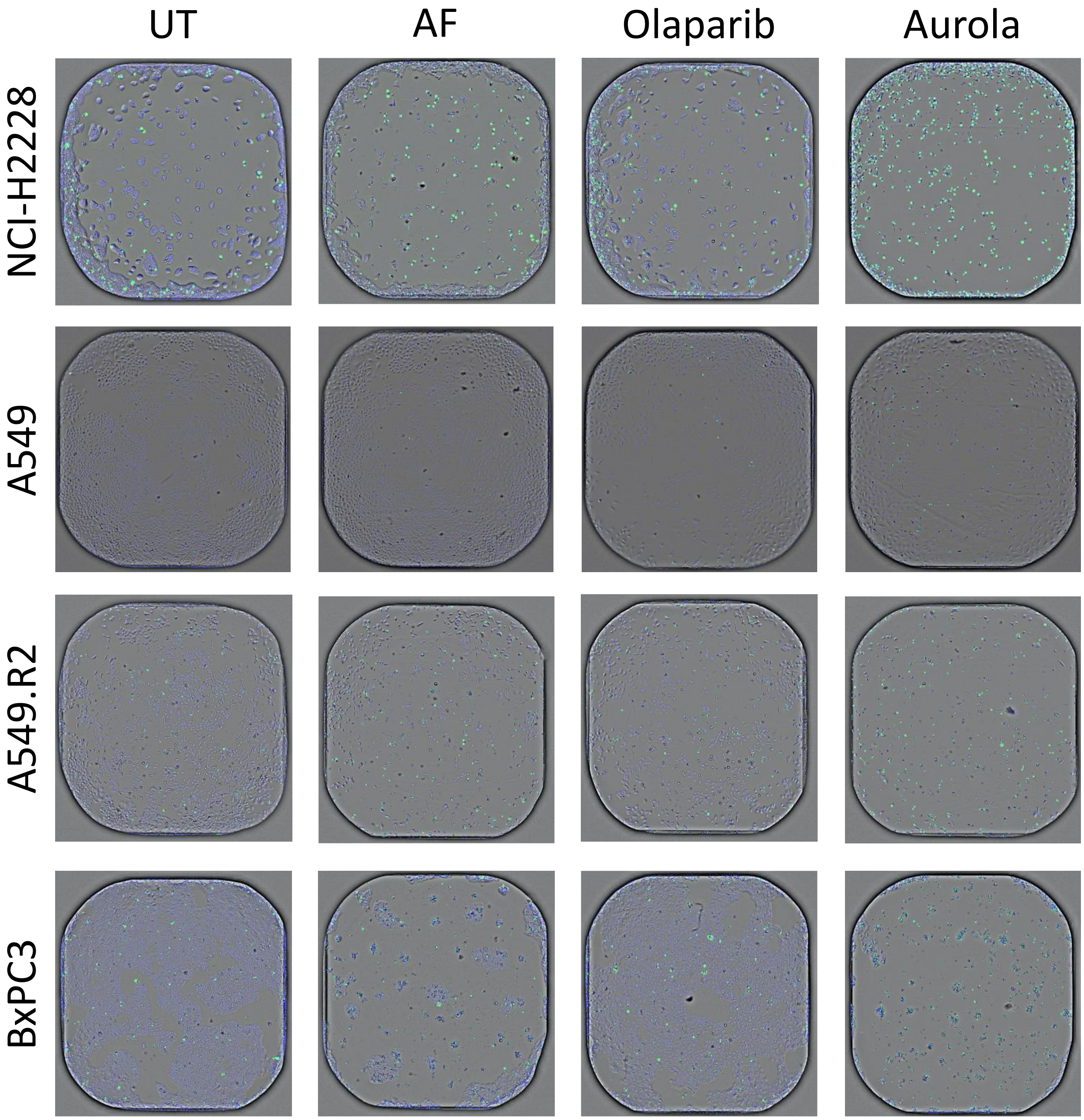

Figure S3. Synergistic interaction between AF and olaparib in NSCLC and PDAC cells *in vitro*. Representative images of NSCLC cell lines NCI-H2888 (6.5 µM AF + 30 µM olaparib), A549 and A549.R2 (5 µM AF + 30 µM olaparib) and the PDAC cell line BxPC3 (2.8 µM AF + 30 µM olaparib) treated with the concentration of AF, olaparib and the aurola combination that resulted in a synergistic interaction after 72 h using the Spark Cyto imaging system. Hoechst signal (blue) visualizes living cells. Cytotox green signal (green) visualizes dead cells.

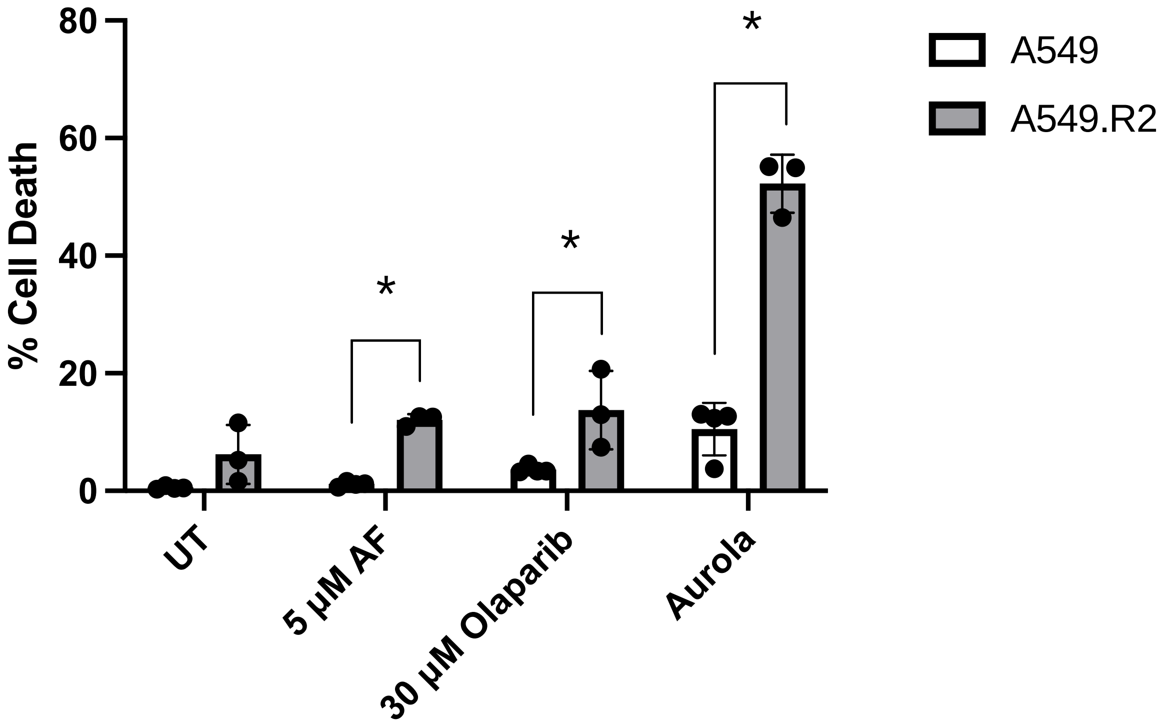

Figure S4. Effect of p53 status on cytotoxic response of AF and olaparib. Difference in the percentage of cell death between WT p53 cell line A549 (white bars) and the mutant p53 cell line A549.R2 (grey bars) after 72 h of treatment with olaparib (30 µM) and AF monotherapy (5 µM) and their combination aurola. Experiments were performed in triplicate. Error bars represent the standard deviation. *$p \leq 0.05$ significant differences between two cell lines.

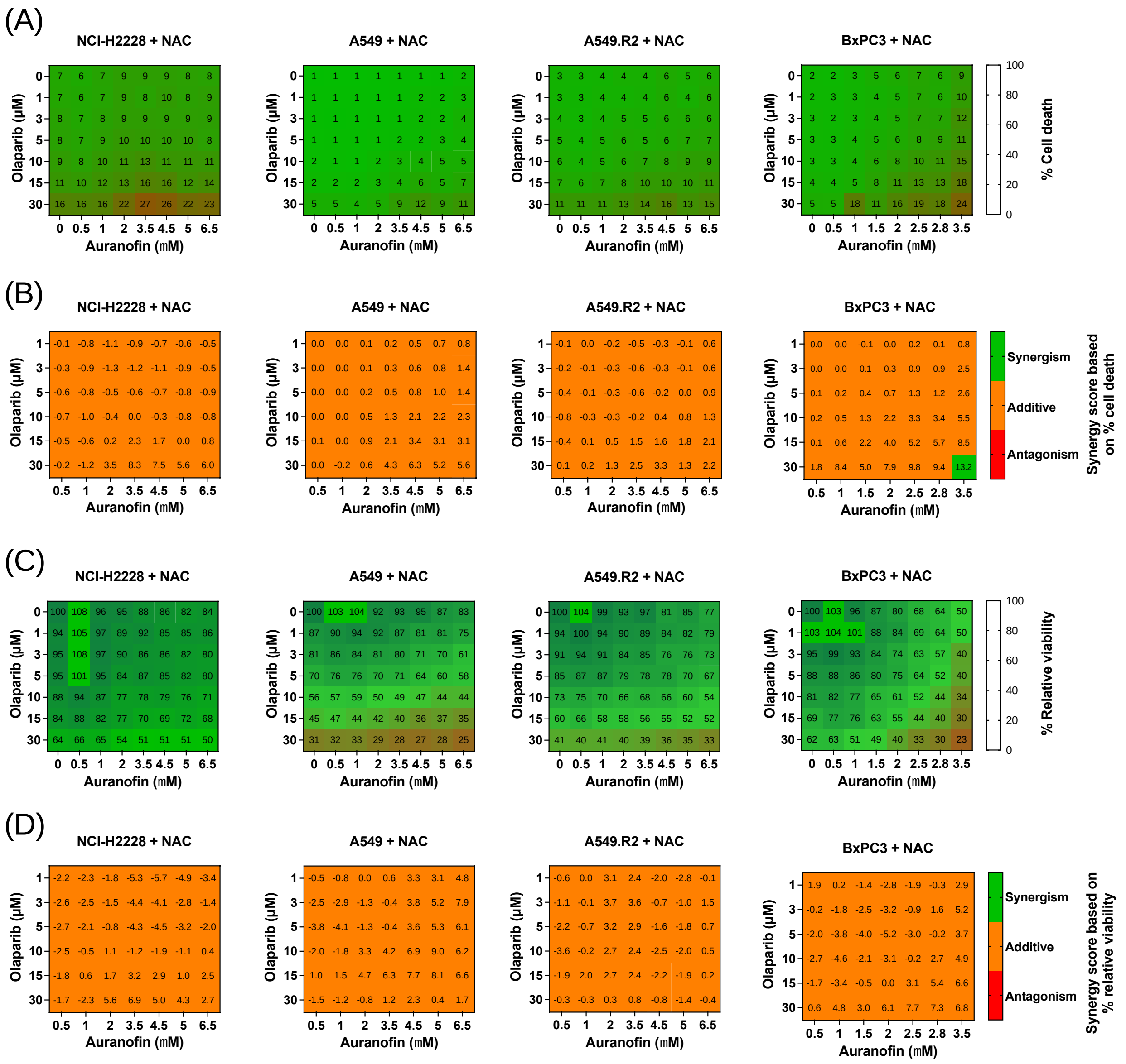

Figure S5. The effect of the ROS scavenger NAC on aurola-mediated cytotoxicity in NSCLC and PDAC cell lines *in vitro*. (**A**) Heatmap representing the cytotoxic effect of AF (0–6.5 µM) and olaparib (0–30 µM) monotherapy and their combinations in the presence of the ROS scavenger NAC (5 mM) after 72 h in the NSCLC cell lines NCI-H2888, A549 and A549.R2 and the PDAC cell line BxPC3. (**B**) Synergy scores for each concentration of AF and olaparib in the presence of the ROS scavenger NAC (5 mM) after 72 h of treatment in the NSCLC cell lines NCI-H2888, A549 and A549.R2 and the PDAC cell line BxPC3. Scores were based on drug combination response normalized to percentage inhibition (cell death). Synergy score > 10: synergistic interaction (green). Synergy score -10 to 10: additive interaction (orange). Synergy score < -10: antagonistic interaction (red) between two drugs. (**C**) Heatmap representing the relative viability of NSCLC cell lines NCI-H2888, A549 and A549.R2 and the PDAC cell line BxPC3 after 72 h of treatment with AF (0–6.5 µM) and olaparib (0–30 µM) monotherapy and their combinations in the presence of the ROS scavenger NAC (5 mM). (**D**) Synergy scores for each concentration of AF and olaparib in the

presence of the ROS scavenger NAC (5 mM) after 72 h of treatment in the NSCLC cell lines NCI-H2888, A549 and A549.R2 and the PDAC cell line BxPC3. Scores were based on drug combination response normalized to percentage viability. Synergy score > 10: synergistic interaction (green). Synergy score -10 to 10: additive interaction (orange). Synergy score < -10: antagonistic interaction (red) between two drugs.

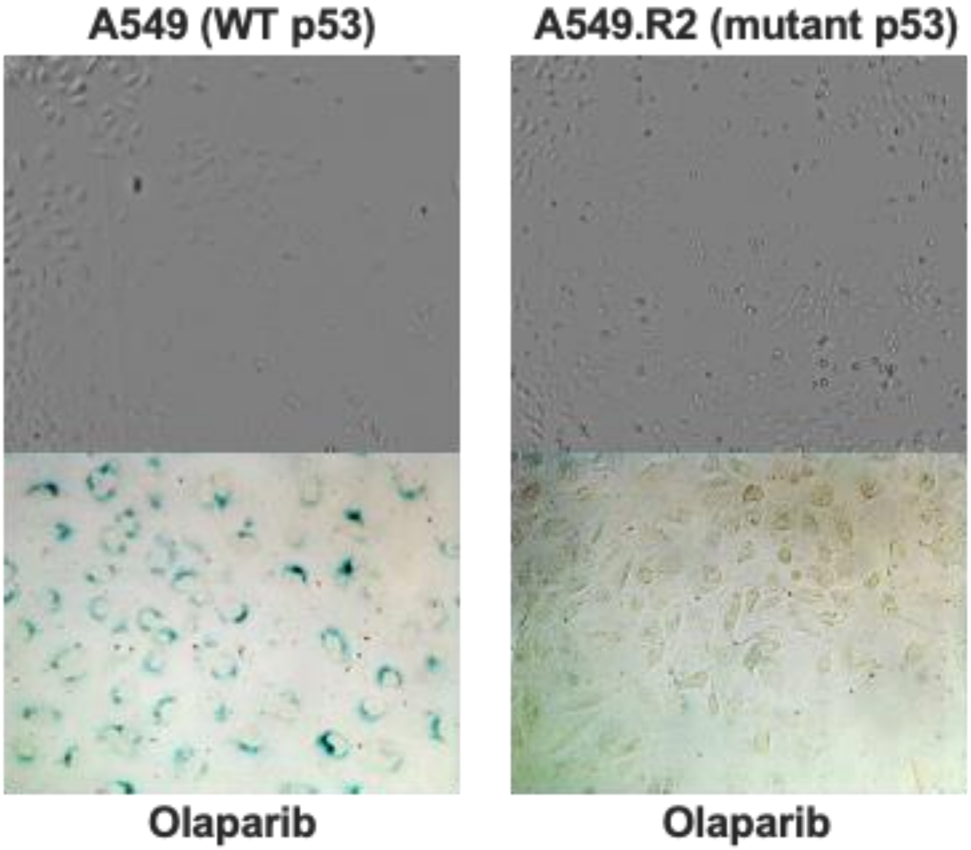

Figure S6. Induction of cellular senescence after treatment with olaparib in WT p53 A549 cells. Visual comparison of β-galactosidase positive stained cells (blue staining) between WT p53 A549 and mutant p53 A549.R2 cells after olaparib treatment (30 μM) for 72 h.

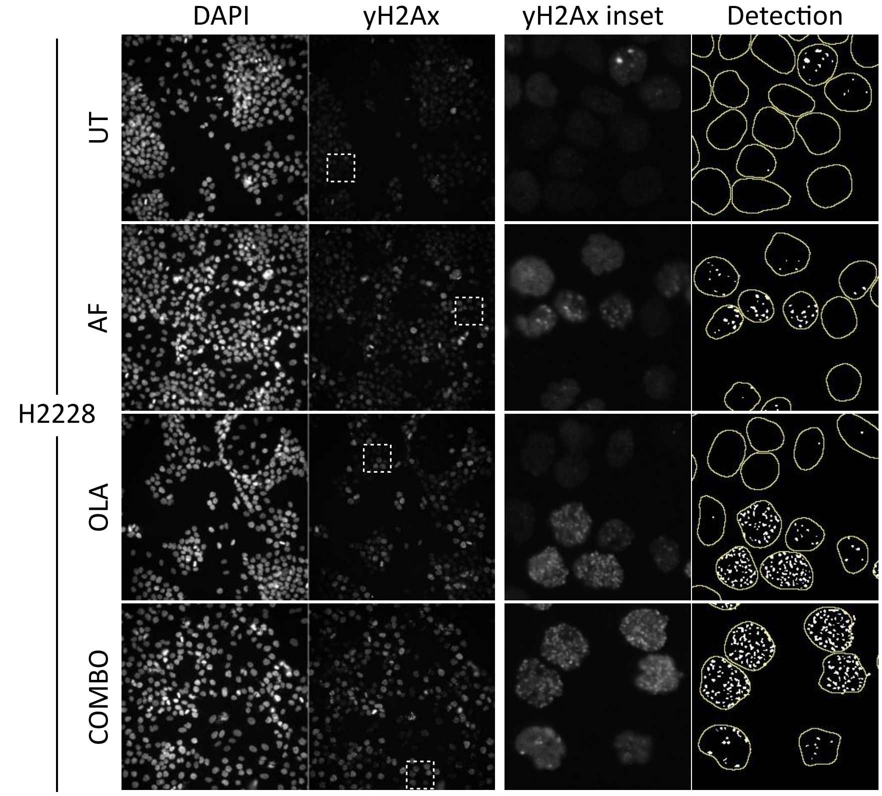
DAPI
yH2Ax
yH2Ax inset
Detection
H2228
UT
AF
OLA
COMBO

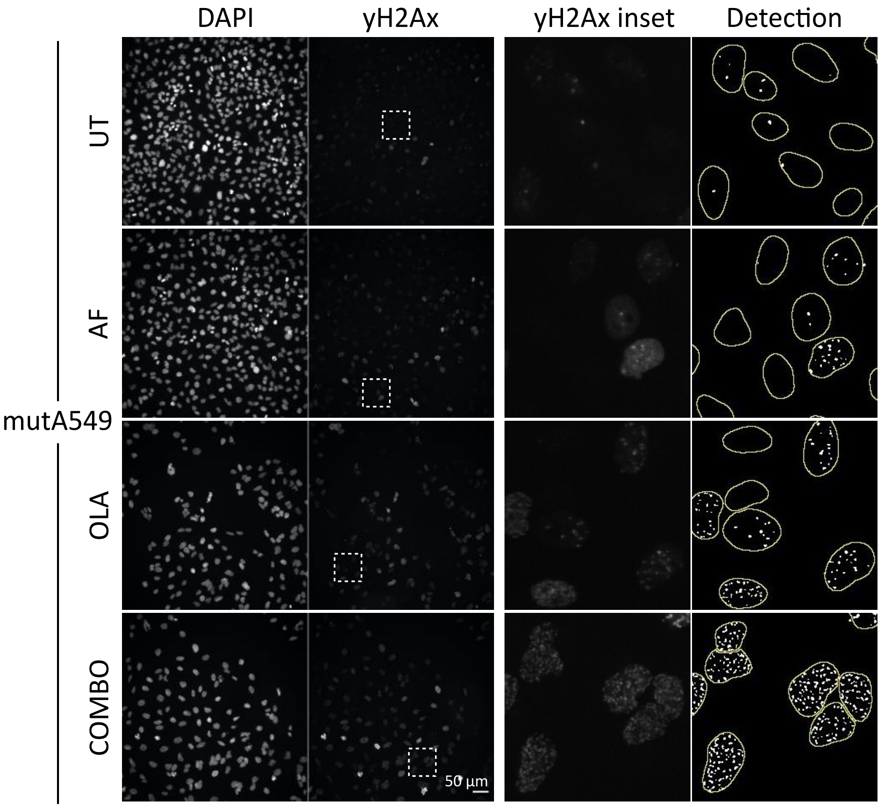
DAPI
yH2Ax
yH2Ax inset
Detection
mutA549
UT
AF
OLA
COMBO
50 µm

Figure S7. Double-stranded break detection in mutant p53 NSCLC cell lines NCI-H2228 and A549.R2. Representative images of untreated (UT), 5 µM AF-, 30 µM olaparib- and aurola-treated NCI-H2228 and A549.R2 cells were taken using a Nikon Ti fluorescence microscope fitted with a x20 objective lens. DNA damage foci were visualized by staining for γH2AX, while nuclei were stained with DAPI.

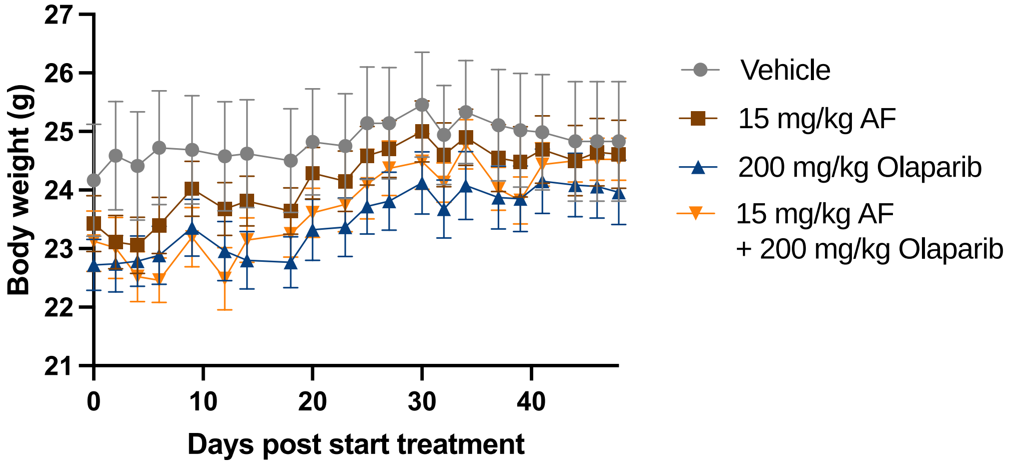

Figure S8. Effect of AF, olaparib and aurola treatment on body weight of 129S2/SvPasCrl (129-) mice. Body weight over time of 129-mice treated for 14 days with vehicle, 15 mg/kg AF, 200 mg/kg olaparib or the combination (15 mg/kg AF + 200 mg/kg olaparib).

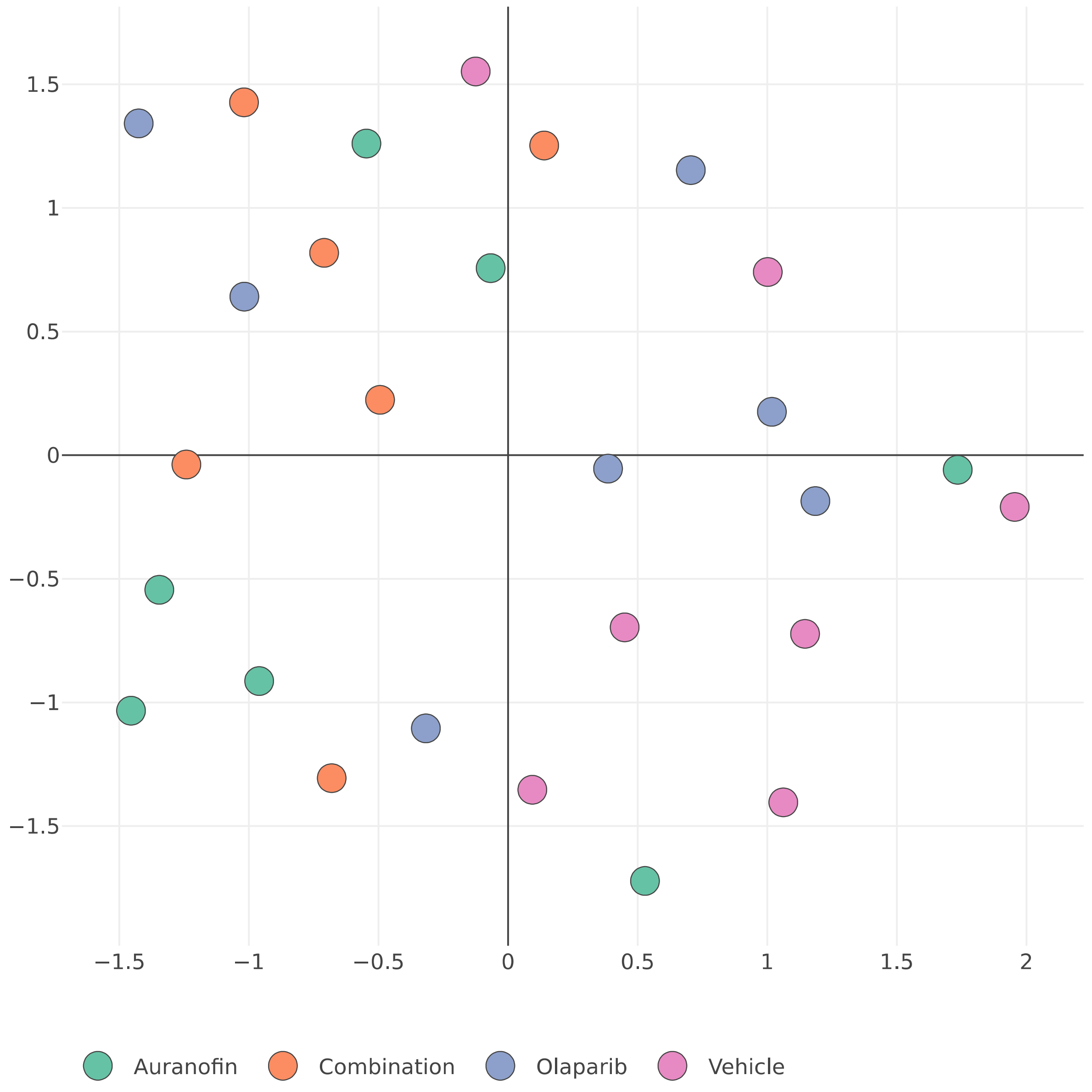

Figure S9. Clustering profile of AF, olaparib and aurola-treated 344SQ tumors. 344SQ tumors (n = 6–8 mice per group) were harvested on day 14 of the treatment schedule for subsequent RNA isolation and sequencing. UMAP-based clustering plot shows unsupervised clustering of the treated mice tumor samples whereby similarity is visualized as proximity of the points. UMAP: Uniform Manifold Approximation and Projection.

(A)

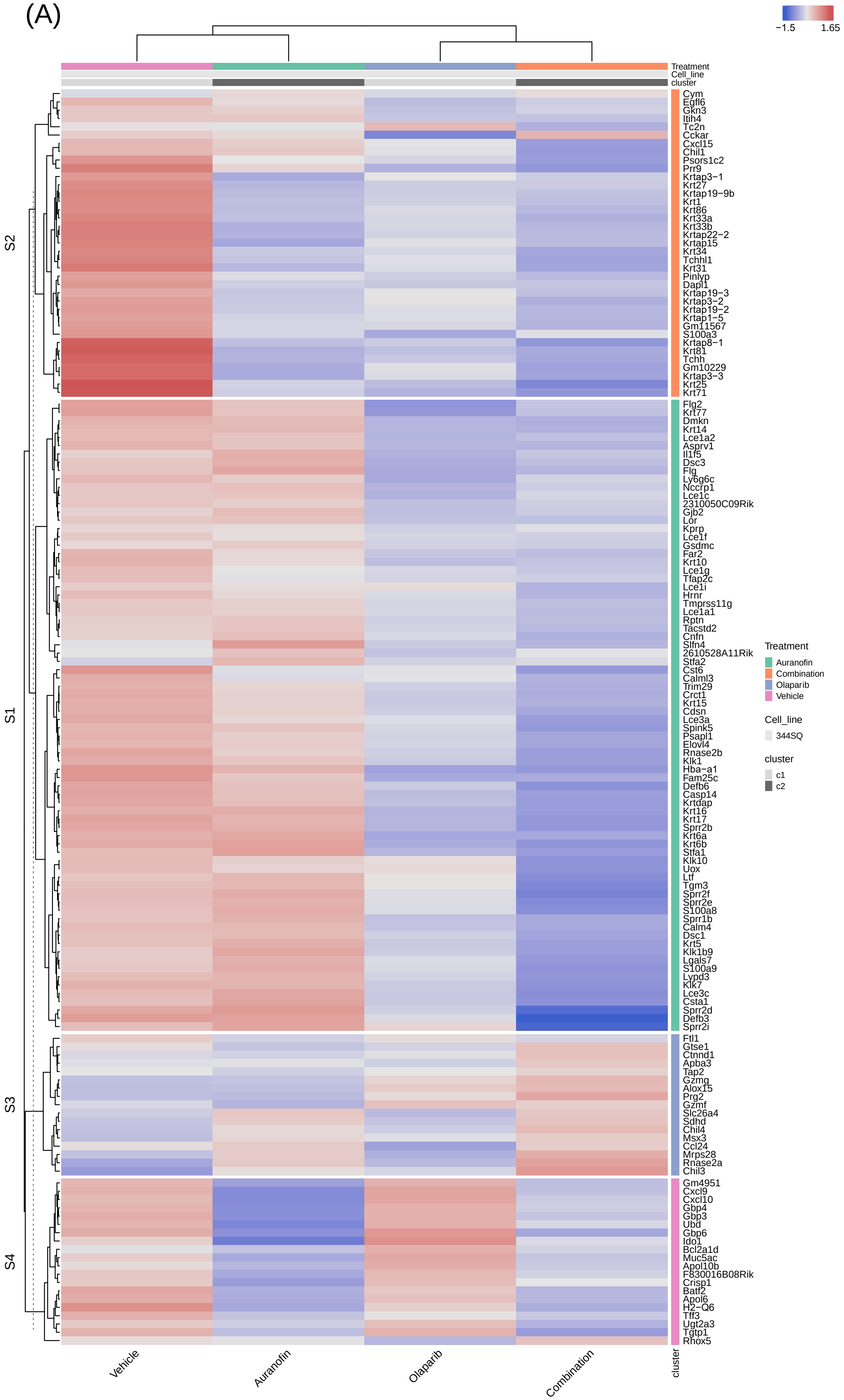

−1.5
1.65
Treatment
Cell_line
cluster
S2
S1
S3
S4
Cym
Egfl6
Gkn3
Itih4
Tc2n
Cckar
Cxcl15
Chil1
Psors1c2
Prr9
Krtap3−1
Krt27
Krtap19−9b
Krt1
Krt86
Krt33a
Krt33b
Krtap22−2
Krtap15
Krt34
Tchhl1
Krt31
Pinlyp
Dapl1
Krtap19−3
Krtap3−2
Krtap19−2
Krtap1−5
Gm11567
S100a3
Krtap8−1
Krt81
Tchh
Gm10229
Krtap3−3
Krt25
Krt71
Flg2
Krt77
Dmkn
Krt14
Lce1a2
Asprv1
Il1f5
Dsc3
Flg
Ly6g6c
Nccrp1
Lce1c
2310050C09Rik
Gjb2
Lor
Kprp
Lce1f
Gsdmc
Far2
Krt10
Lce1g
Tfap2c
Lce1i
Hrnr
Tmprss11g
Lce1a1
Rptn
Tacstd2
Cnfn
Slfn4
2610528A11Rik
Stfa2
Cst6
Calml3
Trim29
Crct1
Krt15
Cdsn
Lce3a
Spink5
Psapl1
Elovl4
Rnase2b
Klk1
Hba−a1
Fam25c
Defb6
Casp14
Krtdap
Krt16
Krt17
Sprr2b
Krt6a
Krt6b
Stfa1
Klk10
Uox
Ltf
Tgm3
Sprr2f
Sprr2e
S100a8
Sprr1b
Calm4
Dsc1
Krt5
Klk1b9
Lgals7
S100a9
Lypd3
Klk7
Lce3c
Csta1
Sprr2d
Defb3
Sprr2i
Ftl1
Gtse1
Ctnnd1
Apba3
Tap2
Gzmg
Alox15
Prg2
Gzmf
Slc26a4
Sdhd
Chil4
Msx3
Ccl24
Mrps28
Rnase2a
Chil3
Gm4951
Cxcl9
Cxcl10
Gbp4
Gbp3
Ubd
Gbp6
Ido1
Bcl2a1d
Muc5ac
Apol10b
F830016B08Rik
Crisp1
Batf2
Apol6
H2−Q6
Tff3
Ugt2a3
Tgtp1
Rhox5
Vehicle
Auranofin
Olaparib
Combination
cluster
Treatment
Auranofin
Combination
Olaparib
Vehicle
Cell_line
344SQ
cluster
c1
c2

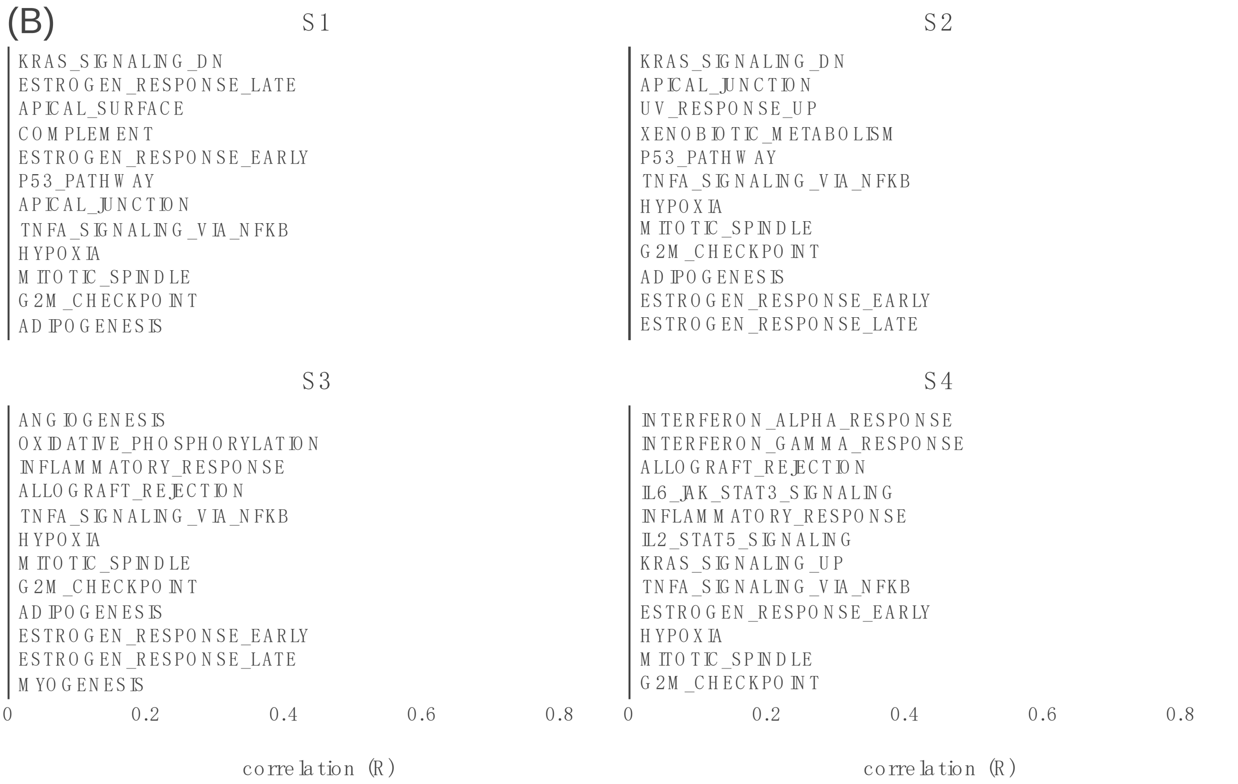

Figure S10. Hierarchical clustering of AF, olaparib and aurola-treated mice tumors on gene level. 344SQ tumors (n = 6–8 mice per group) were harvested on day 14 of the treatment schedule for subsequent RNA isolation and sequencing. (**A**) Clustered heatmap showing gene expression sorted by 2-way hierarchical clustering of the top 150 differentially expressed genes split by treatment group. Red corresponds to overexpression, blue to under expression of the gene. At the same time, gene clusters are functionally annotated in the 'Annotate clusters' of B. (**B**) Cluster annotation for top ranked annotation features (by correlation) for each gene cluster as defined in the heatmap of A. For each cluster, the Annotate cluster section provides a functional annotation using more than 42 published reference databases, including but not limited to well-known databases such as MSigDB, KEGG, and GO.

4. SUPPLEMENTARY WESTERN BLOTS

Original western blot – p53 protein expression in the isogenic NCI-H1299 cell lines

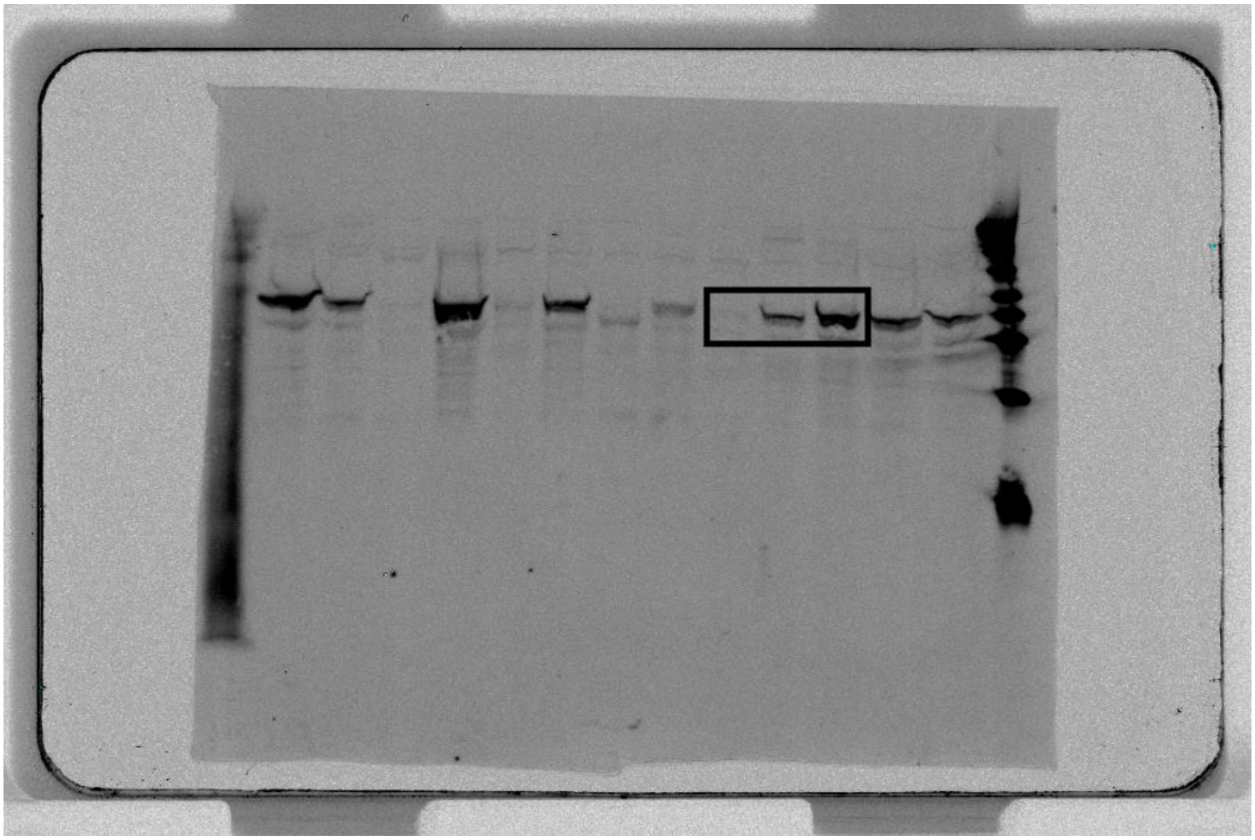

Original western blot – p53 expression in the NCI-H2228 non-template control and shRNA1-3 cells

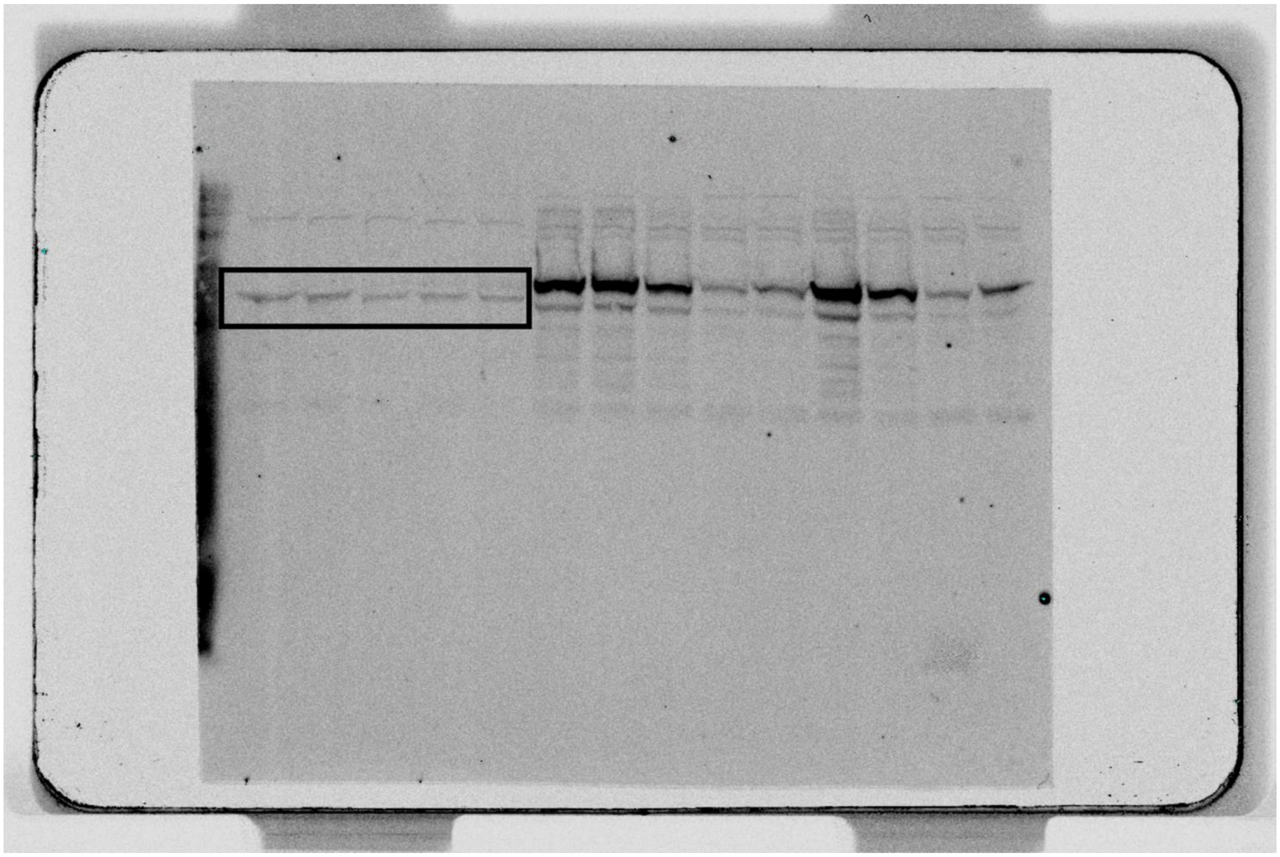

Original western blot –β-actin expression in the NCI-H2228 non-template control and shRNA1-3 cells

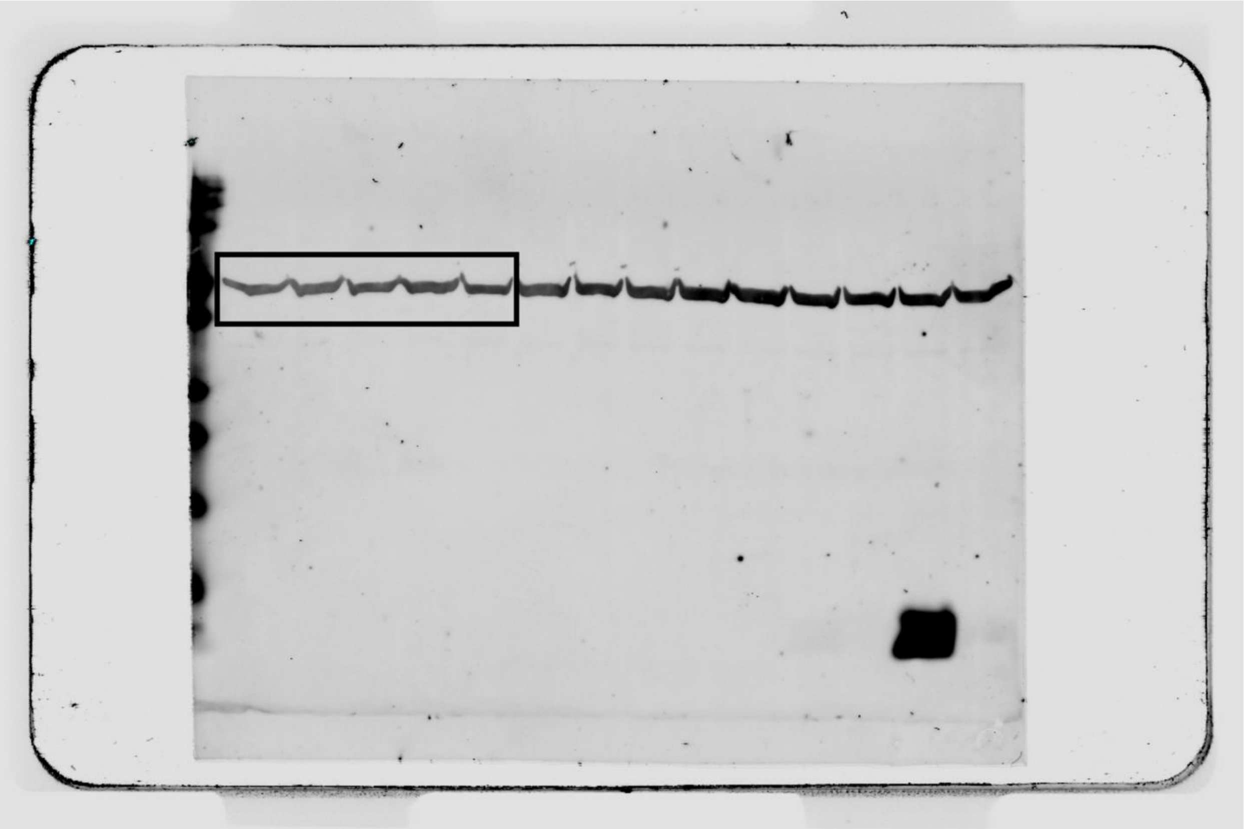

Supplement: Supplementary file 1 [file antioxidants-12-00667-s001.zip › antioxidants-2227758-supplementary.pdf]
